# Supplementary figures and images for: Nitrogen use efficiency in bread wheat: Genetic variation and prospects for improvement
Source: PLoS One. 2024 Apr 10;19(4):e0294755. doi: 10.1371/journal.pone.0294755 (PMC11006162; doi:10.1371/journal.pone.0294755)

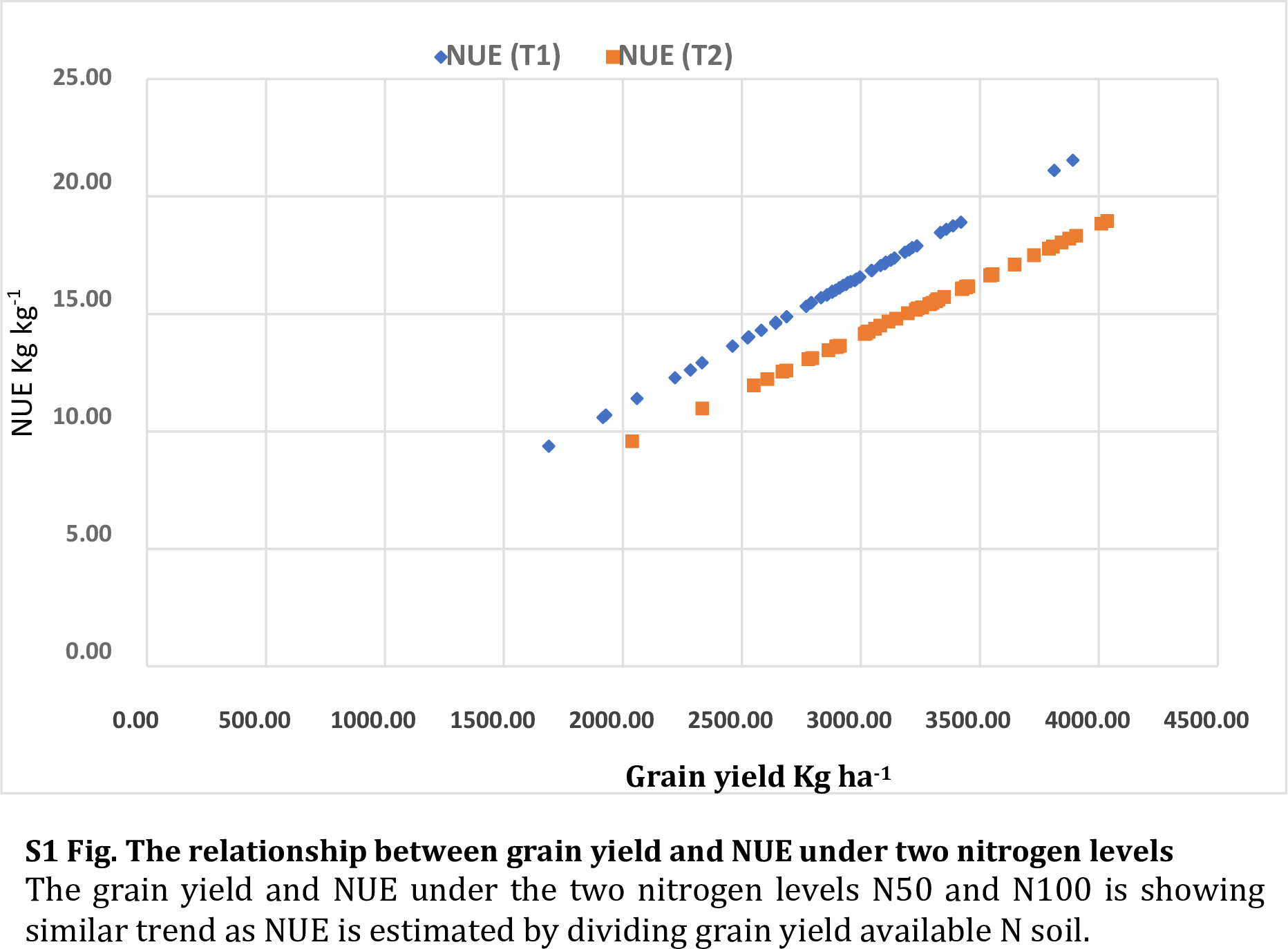

Supplement: S1 Fig — The grain yield and NUE under the two nitrogen levels N50 and N100 is showing similar trend as NUE is estimated by dividing grain yield available N soil. (TIF) [file pone.0294755.s001.tif]

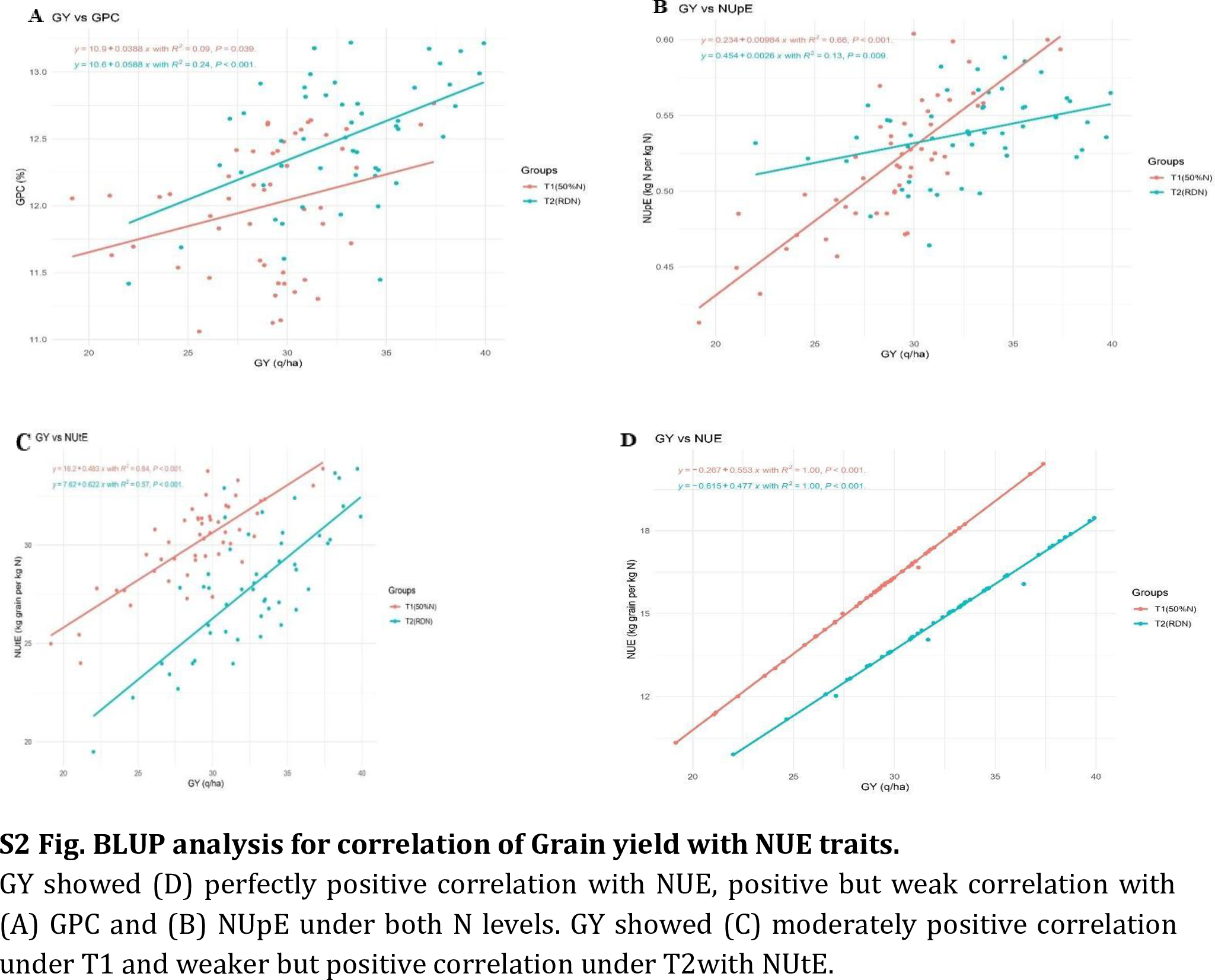

Supplement: S2 Fig — GY showed (D) perfectly positive correlation with NUE, positive but weak correlation with (A) GPC and (B) NUpE under both N levels. GY showed (C) moderately positive correlation under T1 and weaker but positive correlation under T2 with NUtE. (TIF) [file pone.0294755.s002.tif]

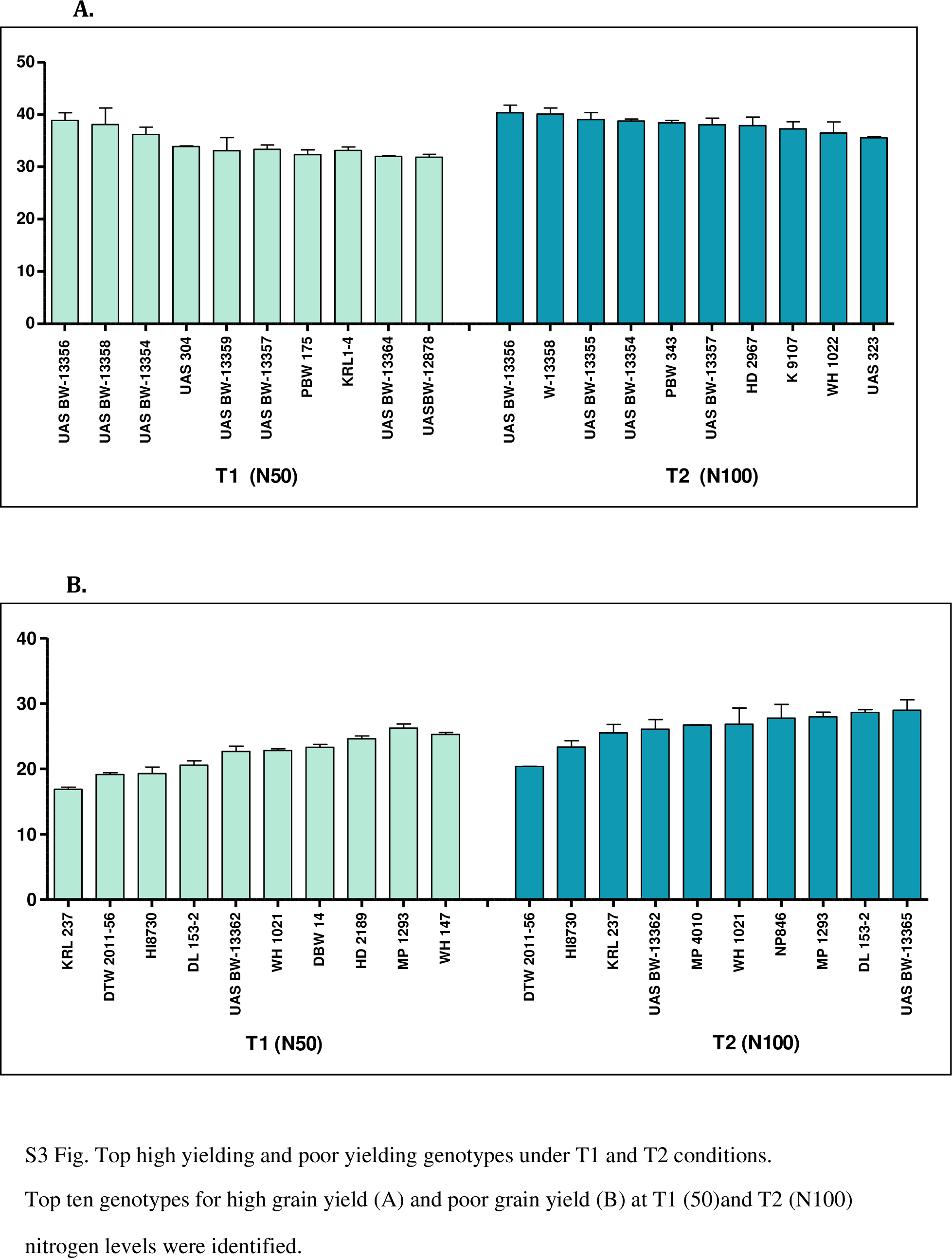

Supplement: S3 Fig — Top ten genotypes for high grain yield (A) and poor grain yield (B) at T1(N50) and T2 (N100) nitrogen levels were identified. (TIF) [file pone.0294755.s003.tif]

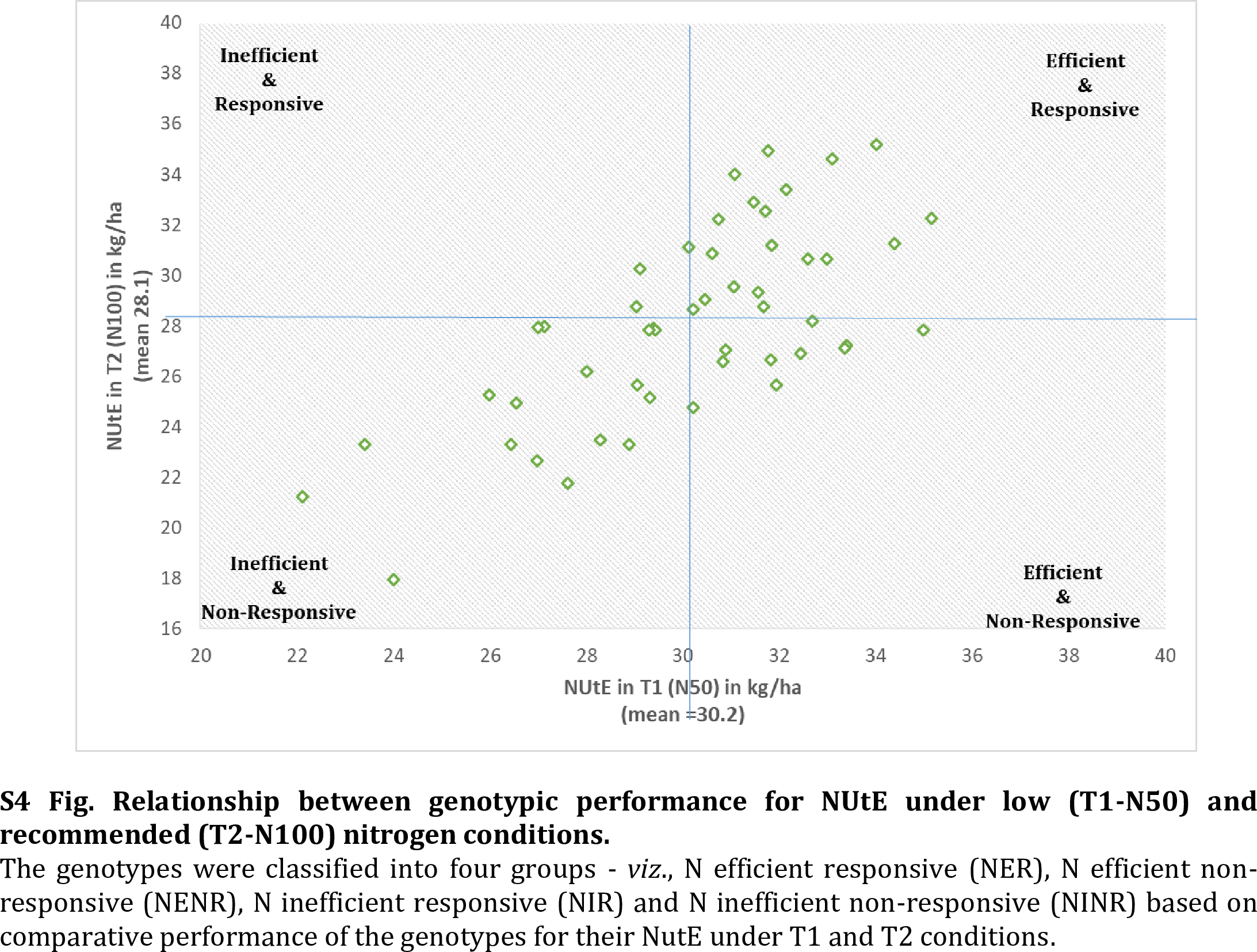

Supplement: S4 Fig — The genotypes were classified into four groups—viz., N efficient responsive (NER), N efficient non-responsive (NENR), N inefficient responsive (NIR) and N inefficient non-responsive (NINR) based on comparative performance of the genotypes for their NutE under T1 and T2 conditions. (TIF) [file pone.0294755.s004.tif]

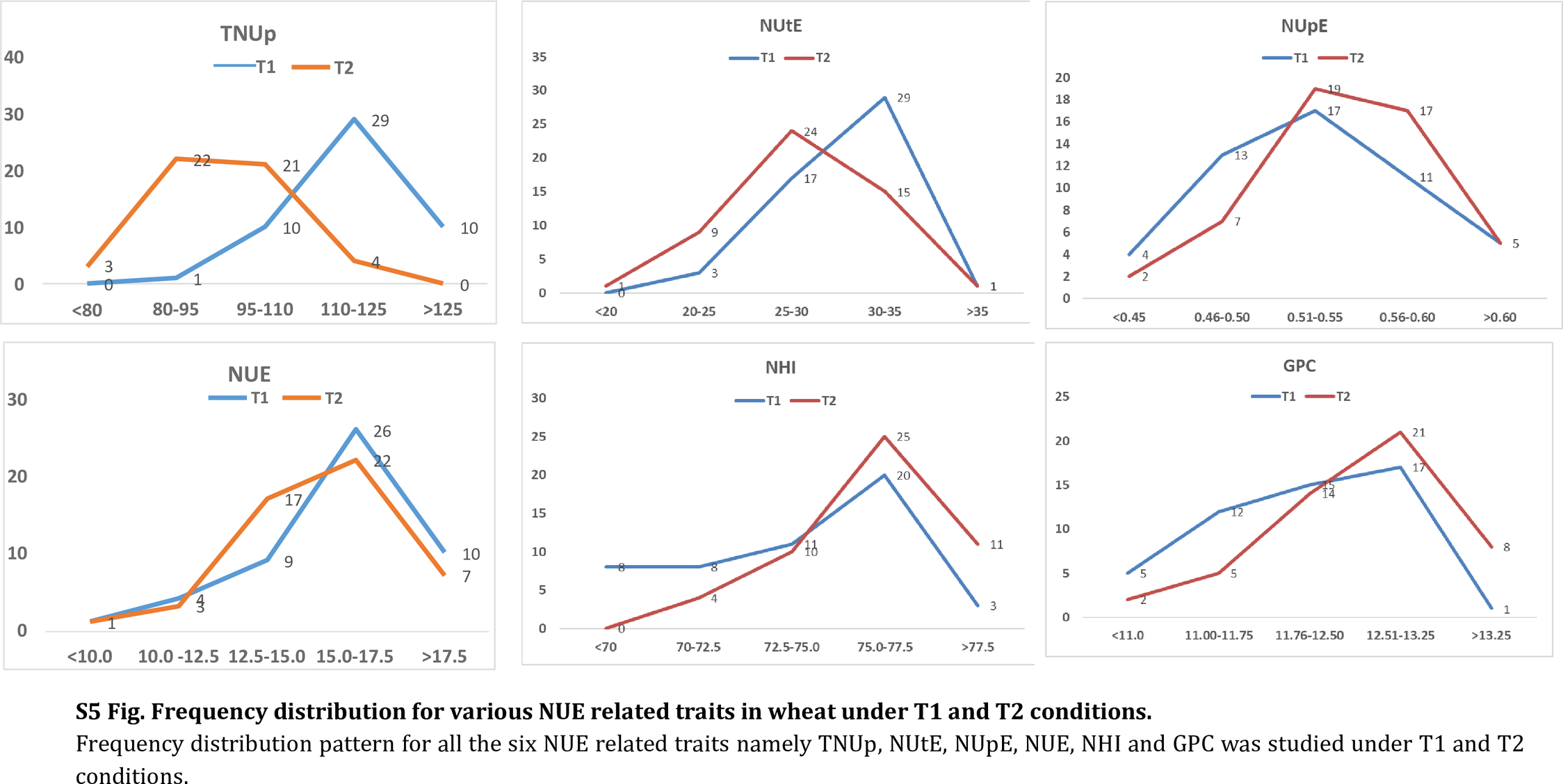

Supplement: S5 Fig — Frequency distribution pattern for all the six NUE related traits namely TNUp, NUtE, NUpE, NUE, NHI and GPC was studied under T1 and T2 conditions. (TIF) [file pone.0294755.s005.tif]
