# Supplementary material for: Nitrogen use efficiency in bread wheat: Genetic variation and prospects for improvement
Source: PLoS One. 2024 Apr 10;19(4):e0294755. doi: 10.1371/journal.pone.0294755 (PMC11006162; doi:10.1371/journal.pone.0294755)
Supplement: S1 Table — DH: Days to Heading, DM: Days to maturity, PH: Plant height (cm), NPT: Number of productive tiller per meter, SL: Spike length (cm), AL: Awn length (cm), SPS: spikelet’s per spike, GPS: grains per spike, TSW: Thousand seed weight (g), GY: Grain yield (q ha-1), BMY: Biomass yield (q ha-1), HI: Harvest index (%), GPC: Grain protein content (%), CC-1: Chlorophyll content at booting stage, NDVI -1: NDVI at booting stage, CC-2: Chlorophyll content at anthesis stage, NDVI- 2: NDVI at anthesis stage, CC-3: Chlorophyll content at grain filling stage, NDVI -3: NDVI at grain filling stage, NHI: Nitrogen harvest index, TNUp: Total nitrogen uptake (kg N ha-1), NUpE: Nitrogen uptake efficiency (kg N kg-1N), NUtE: Nitrogen utilization efficiency (kg grain kg-1N) and NUE: Nitrogen use efficiency (kg grain kg-1N). (DOCX) [file pone.0294755.s006.docx]

**S1 Table. Mean Performance of bread wheat genotypes for agro-morphological, physiological and NUE traits under T1 (soil N + 50 kg Nha^-1^) and T2 (Soil N + 100 kg Nha^-1^) conditions**

|  |  | **Agro-morphological traits** | | | | | | | | | | | | | | | | | | | | | | | | | |
| --- | --- | --- | --- | --- | --- | --- | --- | --- | --- | --- | --- | --- | --- | --- | --- | --- | --- | --- | --- | --- | --- | --- | --- | --- | --- | --- | --- |
| **S. No** | **Genotypes** | **DH** | | **DM** | | **PH** | | **TPM** | | **SL** | | **AL** | | **SPS** | | **GPS** | | **TSW** | | **GY** | | **BMY** | | **HI** | | **GPC** | |
|  |  | **T1** | **T2** | **T1** | **T2** | **T1** | **T2** | **T1** | **T2** | **T1** | **T2** | **T1** | **T2** | **T1** | **T2** | **T1** | **T2** | **T1** | **T2** | **T1** | **T2** | **T1** | **T2** | **T1** | **T2** | **T1** | **T2** |
| 1 | DBW 14 | 51 | 55 | 82 | 93 | 67.48 | 71.04 | 63 | 113 | 7.09 | 8.06 | 6.36 | 7.13 | 14 | 15 | 41 | 43 | 33.84 | 36.55 | 23.32 | 30.28 | 84.62 | 102.94 | 27.70 | 29.44 | 11.33 | 13.17 |
| 2 | DL 153-2 | 63 | 62 | 96 | 99 | 69.85 | 83.78 | 60 | 90 | 6.96 | 8.63 | 6.72 | 4.89 | 15 | 17 | 42 | 47 | 31.48 | 35.33 | 20.60 | 28.64 | 82.03 | 94.44 | 25.14 | 30.39 | 11.58 | 11.61 |
| 3 | DTW 2011-56 | 64 | 63 | 97 | 100 | 90.70 | 88.49 | 64 | 81 | 6.84 | 7.64 | 5.67 | 6.27 | 13 | 17 | 38 | 47 | 32.60 | 34.78 | 19.16 | 20.40 | 89.27 | 120.92 | 21.42 | 16.88 | 12.19 | 10.85 |
| 4 | GW 2013-540 | 58 | 63 | 89 | 97 | 80.20 | 79.23 | 78 | 98 | 7.02 | 7.72 | 6.46 | 7.47 | 15 | 17 | 43 | 48 | 35.28 | 37.28 | 28.32 | 30.33 | 88.25 | 102.71 | 32.09 | 30.19 | 11.41 | 13.06 |
| 5 | GW 322 | 60 | 63 | 92 | 100 | 77.07 | 79.97 | 88 | 90 | 7.80 | 7.31 | 7.07 | 6.38 | 17 | 18 | 49 | 51 | 32.00 | 34.25 | 28.78 | 31.98 | 76.71 | 88.31 | 37.79 | 36.29 | 13.04 | 13.23 |
| 6 | HD 2189 | 59 | 63 | 89 | 100 | 75.83 | 75.44 | 79 | 89 | 7.98 | 8.20 | 6.29 | 5.60 | 18 | 19 | 49 | 54 | 31.05 | 32.35 | 24.60 | 29.03 | 97.46 | 110.81 | 25.27 | 26.51 | 10.56 | 11.56 |
| 7 | HD 2733 | 62 | 66 | 98 | 105 | 73.88 | 88.12 | 95 | 116 | 7.57 | 7.66 | 7.14 | 6.79 | 17 | 18 | 49 | 51 | 35.80 | 36.40 | 29.77 | 35.54 | 93.10 | 123.54 | 32.00 | 28.83 | 12.84 | 12.78 |
| 8 | HD 2967 | 61 | 63 | 96 | 98 | 76.93 | 78.64 | 97 | 102 | 8.70 | 8.18 | 7.34 | 6.70 | 19 | 19 | 54 | 54 | 41.83 | 41.63 | 31.25 | 37.90 | 72.94 | 82.08 | 42.85 | 46.67 | 13.07 | 13.45 |
| 9 | HI 1500 | 61 | 64 | 96 | 102 | 78.53 | 93.98 | 101 | 106 | 7.43 | 7.93 | 6.82 | 6.09 | 17 | 18 | 48 | 50 | 33.92 | 37.10 | 28.94 | 30.23 | 90.46 | 91.98 | 32.06 | 32.97 | 12.32 | 12.56 |
| 10 | HI8730 | 60 | 61 | 99 | 101 | 77.05 | 92.41 | 71 | 90 | 7.74 | 8.58 | 7.58 | 8.50 | 15 | 17 | 44 | 48 | 31.13 | 33.63 | 19.27 | 23.34 | 88.56 | 118.21 | 21.76 | 19.74 | 11.48 | 11.28 |
| 11 | HPW251 | 56 | 65 | 94 | 102 | 63.73 | 81.37 | 73 | 91 | 8.16 | 9.15 | 7.10 | 6.52 | 18 | 18 | 47 | 49 | 38.02 | 38.05 | 26.88 | 30.61 | 85.11 | 91.96 | 31.62 | 33.47 | 12.74 | 13.33 |
| 12 | K 9107 | 59 | 65 | 92 | 100 | 74.18 | 78.87 | 89 | 101 | 8.68 | 8.00 | 6.30 | 6.32 | 17 | 18 | 48 | 50 | 41.38 | 43.72 | 31.42 | 37.29 | 83.38 | 85.48 | 37.69 | 43.62 | 13.10 | 13.63 |
| 13 | Kalyansona | 65 | 68 | 105 | 111 | 70.50 | 81.83 | 79 | 87 | 7.72 | 7.57 | 6.37 | 6.20 | 15 | 18 | 44 | 49 | 31.60 | 33.70 | 30.83 | 33.26 | 96.16 | 97.83 | 32.04 | 34.06 | 12.98 | 12.97 |
| 14 | KRL 237 | 58 | 62 | 91 | 95 | 61.48 | 89.12 | 79 | 80 | 7.99 | 7.26 | 7.11 | 6.40 | 15 | 17 | 47 | 50 | 33.70 | 35.43 | 16.87 | 25.51 | 81.10 | 101.15 | 20.77 | 25.25 | 12.16 | 12.25 |
| 15 | KRL1-4 | 57 | 64 | 92 | 102 | 67.33 | 80.19 | 94 | 99 | 7.82 | 8.79 | 7.29 | 6.11 | 15 | 18 | 42 | 52 | 33.34 | 36.20 | 32.15 | 34.28 | 104.31 | 116.71 | 30.88 | 29.42 | 11.85 | 12.22 |
| 16 | MP 1293 | 56 | 59 | 94 | 94 | 71.58 | 77.50 | 86 | 90 | 6.97 | 6.92 | 8.24 | 6.30 | 16 | 17 | 44 | 48 | 35.68 | 37.43 | 25.24 | 27.97 | 99.60 | 109.81 | 25.32 | 25.52 | 11.21 | 12.02 |
| 17 | MP 4010 | 55 | 57 | 86 | 92 | 70.05 | 88.46 | 97 | 101 | 7.37 | 7.66 | 6.84 | 7.14 | 15 | 17 | 42 | 49 | 32.05 | 36.05 | 26.42 | 26.72 | 90.85 | 109.90 | 29.03 | 24.39 | 12.42 | 12.17 |
| 18 | NP846 | 55 | 60 | 92 | 96 | 81.95 | 73.67 | 90 | 96 | 8.66 | 7.55 | 7.93 | 7.77 | 16 | 17 | 45 | 48 | 32.35 | 36.85 | 27.90 | 27.80 | 100.41 | 115.81 | 27.86 | 24.07 | 12.72 | 13.22 |
| 19 | PBW 175 | 63 | 64 | 99 | 103 | 74.85 | 65.71 | 92 | 100 | 8.07 | 7.91 | 5.62 | 5.11 | 17 | 18 | 49 | 51 | 32.90 | 37.33 | 32.36 | 30.83 | 99.54 | 127.35 | 32.52 | 24.26 | 12.92 | 13.63 |
| 20 | PBW 343 | 63 | 66 | 98 | 100 | 76.28 | 87.70 | 74 | 102 | 7.90 | 8.23 | 6.82 | 7.51 | 18 | 19 | 49 | 52 | 35.54 | 38.80 | 28.80 | 38.45 | 76.79 | 82.46 | 37.50 | 46.72 | 13.06 | 13.20 |
| 21 | RAJ 1972 | 57 | 65 | 93 | 105 | 68.30 | 83.68 | 111 | 101 | 7.69 | 8.49 | 8.29 | 8.47 | 16 | 17 | 47 | 49 | 32.69 | 35.94 | 29.96 | 32.89 | 108.71 | 125.27 | 27.68 | 26.38 | 12.55 | 13.70 |
| 22 | RAJ 4248 | 54 | 59 | 89 | 99 | 75.48 | 79.25 | 92 | 91 | 8.56 | 8.85 | 6.84 | 8.32 | 18 | 19 | 52 | 53 | 37.00 | 39.13 | 27.93 | 32.93 | 106.88 | 125.98 | 26.13 | 26.28 | 12.32 | 12.76 |
| 23 | UAS 304 | 61 | 65 | 97 | 101 | 77.60 | 78.67 | 83 | 103 | 7.93 | 8.51 | 6.19 | 5.74 | 18 | 19 | 51 | 53 | 35.73 | 37.50 | 33.87 | 34.43 | 100.71 | 101.88 | 33.64 | 33.81 | 11.62 | 12.20 |
| 24 | UAS 323 | 65 | 67 | 105 | 106 | 78.43 | 74.28 | 88 | 99 | 9.02 | 9.96 | 6.16 | 7.73 | 19 | 18 | 54 | 50 | 31.58 | 34.80 | 29.25 | 35.55 | 107.31 | 126.06 | 27.53 | 28.23 | 10.99 | 12.68 |
| 25 | WH 147 | 62 | 63 | 99 | 101 | 81.63 | 83.58 | 90 | 91 | 8.26 | 9.20 | 6.29 | 5.69 | 16 | 19 | 48 | 52 | 32.28 | 34.35 | 25.29 | 30.17 | 85.92 | 79.13 | 29.38 | 38.13 | 11.95 | 11.76 |
| 26 | WH 542 | 59 | 58 | 90 | 94 | 78.05 | 86.99 | 101 | 98 | 8.52 | 8.62 | 6.86 | 7.36 | 17 | 18 | 50 | 52 | 31.85 | 35.30 | 29.58 | 32.32 | 88.21 | 118.46 | 33.53 | 27.31 | 10.70 | 11.67 |
| 27 | WH 1021 | 57 | 58 | 88 | 97 | 69.73 | 71.19 | 71 | 122 | 7.09 | 7.80 | 7.06 | 4.72 | 16 | 17 | 47 | 49 | 31.85 | 37.14 | 22.83 | 26.87 | 86.95 | 91.79 | 26.20 | 29.21 | 12.21 | 12.87 |
| 28 | WH 1022 | 57 | 58 | 92 | 98 | 73.57 | 69.36 | 84 | 98 | 8.20 | 9.28 | 6.83 | 7.95 | 17 | 18 | 50 | 50 | 36.38 | 39.98 | 30.47 | 36.47 | 95.51 | 124.85 | 31.95 | 29.21 | 12.94 | 13.17 |
| 29 | UAS BW-13354 | 62 | 63 | 92 | 103 | 75.33 | 79.80 | 91 | 99 | 8.37 | 7.41 | 7.07 | 6.35 | 17 | 18 | 50 | 50 | 36.15 | 40.08 | 34.20 | 38.76 | 85.47 | 90.52 | 39.95 | 42.94 | 12.52 | 12.95 |
| 30 | UAS BW-13355 | 55 | 61 | 89 | 99 | 77.46 | 72.35 | 99 | 104 | 7.93 | 7.95 | 5.80 | 7.04 | 15 | 18 | 42 | 52 | 35.80 | 38.93 | 29.10 | 39.06 | 84.00 | 93.25 | 34.62 | 41.88 | 12.70 | 13.60 |
| 31 | UAS BW-13356 | 60 | 65 | 97 | 99 | 78.40 | 83.00 | 107 | 103 | 10.02 | 9.68 | 6.91 | 6.63 | 20 | 20 | 58 | 58 | 43.05 | 46.40 | 38.91 | 40.36 | 74.66 | 84.00 | 52.12 | 48.13 | 13.29 | 13.69 |
| 32 | UAS BW-13357 | 58 | 62 | 96 | 102 | 82.00 | 63.98 | 89 | 114 | 8.28 | 8.32 | 6.37 | 6.90 | 17 | 18 | 48 | 51 | 38.03 | 40.73 | 33.34 | 38.08 | 83.50 | 93.33 | 39.95 | 41.16 | 12.75 | 12.59 |
| 33 | UAS BW-13358 | 64 | 66 | 99 | 108 | 78.25 | 79.96 | 101 | 98 | 10.07 | 7.95 | 7.06 | 7.26 | 19 | 20 | 56 | 56 | 40.25 | 44.43 | 38.12 | 40.12 | 75.03 | 80.23 | 50.81 | 50.01 | 13.04 | 13.34 |
| 34 | UAS BW-13359 | 62 | 65 | 100 | 105 | 81.43 | 80.78 | 85 | 87 | 9.38 | 9.68 | 7.10 | 6.83 | 18 | 19 | 51 | 53 | 35.71 | 37.75 | 33.60 | 35.43 | 93.91 | 114.33 | 35.80 | 31.30 | 12.99 | 12.71 |
| 35 | UAS BW-13360 | 63 | 62 | 97 | 100 | 79.25 | 85.28 | 80 | 93 | 8.02 | 8.52 | 6.65 | 6.56 | 15 | 16 | 43 | 45 | 33.50 | 37.85 | 29.39 | 31.48 | 80.71 | 91.25 | 36.41 | 34.65 | 12.73 | 13.08 |
| 36 | UAS BW-13361 | 60 | 60 | 96 | 100 | 68.75 | 77.91 | 90 | 90 | 7.79 | 8.08 | 6.13 | 6.18 | 17 | 19 | 48 | 52 | 35.65 | 38.89 | 29.09 | 33.50 | 85.30 | 112.71 | 34.11 | 29.75 | 10.67 | 12.86 |
| 37 | UAS BW-13362 | 55 | 56 | 86 | 94 | 68.90 | 82.22 | 67 | 93 | 7.35 | 7.96 | 7.07 | 6.52 | 15 | 16 | 44 | 45 | 34.55 | 36.17 | 22.19 | 26.08 | 92.52 | 128.83 | 24.00 | 20.28 | 12.17 | 12.80 |
| 38 | UAS BW-13363 | 59 | 62 | 95 | 99 | 71.50 | 79.21 | 104 | 112 | 7.30 | 8.65 | 5.95 | 5.69 | 16 | 18 | 44 | 50 | 32.89 | 36.23 | 27.70 | 28.98 | 82.45 | 89.52 | 33.62 | 32.78 | 11.85 | 12.25 |
| 39 | UAS BW-13364 | 62 | 64 | 103 | 101 | 69.20 | 80.75 | 88 | 102 | 8.10 | 9.06 | 6.87 | 6.26 | 17 | 18 | 48 | 49 | 29.13 | 34.05 | 32.02 | 34.53 | 90.22 | 93.44 | 35.51 | 37.15 | 12.04 | 10.90 |
| 40 | UAS BW-13365 | 63 | 66 | 104 | 102 | 74.45 | 87.57 | 65 | 100 | 7.91 | 8.81 | 6.93 | 8.24 | 15 | 18 | 42 | 51 | 31.92 | 33.98 | 25.81 | 28.97 | 87.61 | 103.38 | 29.45 | 28.23 | 11.80 | 12.54 |
| 41 | UAS BW-13366 | 62 | 63 | 97 | 102 | 68.48 | 85.28 | 78 | 100 | 7.90 | 8.84 | 6.32 | 6.42 | 17 | 18 | 47 | 52 | 30.60 | 33.80 | 29.44 | 33.22 | 86.04 | 106.98 | 34.27 | 31.34 | 11.14 | 12.41 |
| 42 | UAS BW-13367 | 56 | 63 | 91 | 98 | 79.33 | 86.17 | 95 | 99 | 7.70 | 8.36 | 6.33 | 6.41 | 17 | 18 | 48 | 51 | 29.58 | 31.58 | 31.05 | 32.40 | 94.61 | 117.79 | 32.87 | 27.84 | 11.18 | 12.97 |
| 43 | UASBW 12758 | 62 | 65 | 99 | 104 | 71.80 | 78.64 | 78 | 102 | 7.96 | 9.18 | 7.03 | 8.20 | 16 | 18 | 46 | 51 | 33.63 | 35.68 | 26.42 | 31.17 | 86.79 | 114.83 | 30.44 | 27.47 | 12.15 | 12.22 |
| 44 | UASBW-10227 | 57 | 60 | 94 | 95 | 67.50 | 70.43 | 93 | 105 | 8.13 | 8.63 | 7.62 | 7.31 | 15 | 19 | 44 | 54 | 33.25 | 36.75 | 28.57 | 29.13 | 98.24 | 113.58 | 29.07 | 25.89 | 12.26 | 11.15 |
| 45 | UASBW10453 | 62 | 64 | 101 | 102 | 79.68 | 81.22 | 78 | 99 | 9.09 | 8.42 | 7.63 | 6.71 | 17 | 17 | 49 | 47 | 34.63 | 38.90 | 30.44 | 35.44 | 108.54 | 121.23 | 28.21 | 30.07 | 11.04 | 12.04 |
| 46 | UASBW-11948 | 59 | 59 | 98 | 99 | 81.73 | 82.37 | 83 | 88 | 7.08 | 8.49 | 6.33 | 7.93 | 16 | 17 | 48 | 47 | 32.73 | 37.40 | 29.73 | 34.42 | 101.16 | 115.77 | 29.30 | 29.74 | 11.27 | 11.77 |
| 47 | UASBW-12380 | 59 | 62 | 97 | 101 | 76.38 | 73.36 | 94 | 80 | 7.63 | 8.37 | 5.58 | 5.55 | 18 | 17 | 49 | 48 | 34.90 | 36.69 | 31.00 | 34.25 | 99.00 | 132.38 | 31.32 | 25.95 | 12.03 | 12.13 |
| 48 | UASBW-12876 | 59 | 63 | 95 | 105 | 73.68 | 70.61 | 102 | 81 | 7.63 | 7.46 | 5.74 | 7.30 | 15 | 17 | 42 | 49 | 32.10 | 36.13 | 29.80 | 33.01 | 95.36 | 97.25 | 31.24 | 33.93 | 11.14 | 12.42 |
| 49 | UASBW-12877 | 60 | 62 | 93 | 100 | 67.75 | 74.90 | 72 | 83 | 7.12 | 8.00 | 5.43 | 6.23 | 15 | 18 | 42 | 51 | 32.90 | 36.58 | 28.58 | 32.57 | 94.49 | 109.77 | 30.23 | 29.73 | 11.36 | 12.58 |
| 50 | UASBW-12878 | 58 | 59 | 94 | 98 | 67.00 | 82.72 | 97 | 114 | 7.34 | 7.66 | 6.77 | 6.53 | 18 | 18 | 51 | 50 | 29.22 | 33.43 | 31.84 | 33.16 | 100.00 | 122.88 | 32.12 | 27.03 | 10.95 | 12.13 |

| **S.No** | **Genotypes** | **Physiological traits** | | | | | | | | | | | | **NUE related traits** | | | | | | | | | |
| --- | --- | --- | --- | --- | --- | --- | --- | --- | --- | --- | --- | --- | --- | --- | --- | --- | --- | --- | --- | --- | --- | --- | --- |
|  |  | **Booting stage** | | | | **Anthesis stage** | | | | **Grain filling stage** | | | |  |  |  |  |  |  |  |  |  |  |
|  |  | **CC-1** | | **NDVI-1** | | **CC-2** | | **NDVI-2** | | **CC-3** | | **NDVI-3** | | **NHI** | | **TNUPE** | | **NUPE** | | **NUTE** | | **NUE** | |
|  |  | **T1** | **T2** | **T1** | **T2** | **T1** | **T2** | **T1** | **T2** | **T1** | **T2** | **T1** | **T2** | **T1** | **T2** | **T1** | **T2** | **T1** | **T2** | **T1** | **T2** | **T1** | **T2** |
| 1 | DBW 14 | 45.33 | 51.00 | 0.55 | 0.50 | 44.50 | 57.43 | 0.55 | 0.57 | 40.65 | 50.97 | 0.50 | 0.53 | 70.03 | 73.69 | 89.98 | 120.21 | 0.50 | 0.57 | 25.98 | 25.26 | 12.92 | 14.23 |
| 2 | DL 153-2 | 46.55 | 50.05 | 0.51 | 0.51 | 48.43 | 56.05 | 0.55 | 0.57 | 47.48 | 49.55 | 0.54 | 0.53 | 75.43 | 71.26 | 75.98 | 102.32 | 0.42 | 0.48 | 27.12 | 27.99 | 11.39 | 13.45 |
| 3 | DTW 2011-56 | 44.00 | 49.53 | 0.49 | 0.54 | 42.00 | 55.28 | 0.52 | 0.59 | 40.50 | 48.90 | 0.5 | 0.54 | 76.86 | 66.72 | 79.71 | 113.73 | 0.44 | 0.53 | 24.01 | 17.95 | 10.59 | 9.57 |
| 4 | GW 2013-540 | 44.65 | 48.65 | 0.53 | 0.54 | 45.92 | 54.90 | 0.56 | 0.53 | 46.42 | 48.28 | 0.53 | 0.54 | 75.65 | 67.30 | 87.32 | 115.15 | 0.49 | 0.54 | 32.44 | 26.91 | 15.69 | 14.24 |
| 5 | GW 322 | 50.85 | 52.33 | 0.54 | 0.57 | 52.18 | 58.33 | 0.59 | 0.63 | 49.95 | 51.83 | 0.56 | 0.60 | 76.80 | 77.35 | 90.40 | 102.60 | 0.50 | 0.48 | 31.84 | 31.19 | 15.96 | 15.02 |
| 6 | HD 2189 | 45.18 | 48.60 | 0.56 | 0.52 | 47.00 | 54.60 | 0.56 | 0.58 | 46.43 | 47.68 | 0.55 | 0.54 | 74.66 | 75.00 | 83.74 | 104.17 | 0.47 | 0.49 | 29.39 | 27.92 | 13.63 | 13.61 |
| 7 | HD 2733 | 48.90 | 53.58 | 0.54 | 0.56 | 49.66 | 59.83 | 0.55 | 0.62 | 48.80 | 53.20 | 0.57 | 0.57 | 78.58 | 76.17 | 96.69 | 133.53 | 0.54 | 0.63 | 30.84 | 26.60 | 16.44 | 16.66 |
| 8 | HD 2967 | 52.60 | 56.80 | 0.61 | 0.56 | 53.58 | 62.80 | 0.63 | 0.64 | 53.20 | 55.40 | 0.62 | 0.59 | 74.04 | 68.96 | 95.93 | 124.23 | 0.54 | 0.59 | 32.59 | 30.64 | 17.29 | 17.78 |
| 9 | HI 1500 | 46.65 | 51.38 | 0.53 | 0.51 | 47.95 | 58.38 | 0.56 | 0.58 | 47.85 | 51.38 | 0.55 | 0.55 | 78.35 | 75.21 | 94.21 | 102.17 | 0.53 | 0.48 | 30.73 | 32.22 | 16.01 | 14.17 |
| 10 | HI8730 | 44.00 | 49.75 | 0.52 | 0.51 | 48.80 | 55.75 | 0.53 | 0.58 | 44.60 | 49.25 | 0.54 | 0.54 | 71.49 | 71.65 | 87.41 | 109.87 | 0.49 | 0.52 | 22.11 | 21.24 | 10.68 | 10.97 |
| 11 | HPW251 | 48.33 | 50.98 | 0.52 | 0.54 | 51.20 | 56.98 | 0.54 | 0.61 | 50.00 | 50.73 | 0.57 | 0.57 | 76.79 | 78.29 | 92.38 | 101.10 | 0.51 | 0.48 | 29.11 | 30.28 | 14.88 | 14.37 |
| 12 | K 9107 | 54.43 | 56.93 | 0.56 | 0.57 | 55.75 | 62.93 | 0.62 | 0.63 | 54.33 | 56.43 | 0.58 | 0.58 | 73.88 | 73.16 | 104.26 | 119.87 | 0.58 | 0.57 | 30.13 | 31.11 | 17.38 | 17.49 |
| 13 | Kalyansona | 46.73 | 47.78 | 0.55 | 0.57 | 48.73 | 53.78 | 0.58 | 0.62 | 47.98 | 47.53 | 0.57 | 0.59 | 78.28 | 75.78 | 102.01 | 116.11 | 0.57 | 0.55 | 30.21 | 28.66 | 17.05 | 15.62 |
| 14 | KRL 237 | 46.78 | 51.88 | 0.52 | 0.52 | 48.88 | 58.13 | 0.6 | 0.58 | 48.30 | 51.50 | 0.51 | 0.55 | 76.47 | 71.19 | 71.86 | 109.35 | 0.40 | 0.51 | 23.41 | 23.32 | 9.36 | 11.96 |
| 15 | KRL1-4 | 45.50 | 47.15 | 0.55 | 0.53 | 47.95 | 53.65 | 0.55 | 0.60 | 47.88 | 46.90 | 0.55 | 0.57 | 77.50 | 76.60 | 103.53 | 116.13 | 0.58 | 0.54 | 31.06 | 29.54 | 17.81 | 16.08 |
| 16 | MP 1293 | 47.15 | 52.03 | 0.52 | 0.54 | 48.15 | 56.98 | 0.6 | 0.60 | 48.13 | 51.00 | 0.54 | 0.57 | 76.68 | 70.57 | 89.22 | 119.08 | 0.50 | 0.56 | 28.29 | 23.49 | 13.99 | 13.12 |
| 17 | MP 4010 | 45.25 | 50.43 | 0.49 | 0.51 | 47.05 | 56.43 | 0.52 | 0.57 | 45.65 | 49.93 | 0.51 | 0.54 | 76.22 | 69.40 | 95.57 | 122.72 | 0.53 | 0.58 | 27.61 | 21.78 | 14.60 | 12.54 |
| 18 | NP846 | 43.55 | 49.25 | 0.51 | 0.52 | 45.20 | 55.50 | 0.56 | 0.58 | 43.70 | 48.88 | 0.54 | 0.56 | 76.42 | 75.25 | 105.49 | 119.26 | 0.59 | 0.56 | 26.44 | 23.32 | 15.46 | 13.07 |
| 19 | PBW 175 | 49.23 | 53.43 | 0.55 | 0.54 | 51.03 | 59.68 | 0.55 | 0.58 | 50.05 | 53.05 | 0.54 | 0.56 | 75.61 | 76.46 | 111.89 | 132.13 | 0.62 | 0.62 | 28.89 | 23.31 | 17.89 | 14.49 |
| 20 | PBW 343 | 48.00 | 54.13 | 0.58 | 0.50 | 51.38 | 60.13 | 0.59 | 0.60 | 49.70 | 53.38 | 0.56 | 0.55 | 77.92 | 76.16 | 90.65 | 110.20 | 0.51 | 0.52 | 31.77 | 34.92 | 15.93 | 18.04 |
| 21 | RAJ 1972 | 49.05 | 55.38 | 0.58 | 0.54 | 51.13 | 61.18 | 0.64 | 0.61 | 50.33 | 54.25 | 0.61 | 0.57 | 76.25 | 75.42 | 113.11 | 131.68 | 0.63 | 0.62 | 26.55 | 24.96 | 16.57 | 15.41 |
| 22 | RAJ 4248 | 48.60 | 53.53 | 0.53 | 0.56 | 48.73 | 59.53 | 0.57 | 0.62 | 49.45 | 53.03 | 0.55 | 0.58 | 78.29 | 75.95 | 99.69 | 126.39 | 0.56 | 0.59 | 28.01 | 26.21 | 15.48 | 15.42 |
| 23 | UAS 304 | 46.53 | 47.83 | 0.56 | 0.54 | 47.75 | 53.83 | 0.59 | 0.60 | 47.38 | 46.83 | 0.55 | 0.57 | 76.19 | 74.44 | 102.70 | 112.41 | 0.57 | 0.53 | 32.98 | 30.64 | 18.74 | 16.15 |
| 24 | UAS 323 | 50.25 | 51.50 | 0.52 | 0.56 | 50.26 | 57.75 | 0.58 | 0.62 | 43.95 | 50.63 | 0.53 | 0.58 | 76.67 | 77.32 | 95.82 | 122.52 | 0.53 | 0.58 | 30.46 | 29.04 | 16.22 | 16.68 |
| 25 | WH 147 | 42.95 | 47.68 | 0.54 | 0.49 | 44.38 | 53.18 | 0.59 | 0.54 | 44.68 | 46.93 | 0.56 | 0.51 | 77.64 | 74.18 | 81.29 | 88.97 | 0.45 | 0.42 | 31.08 | 34.01 | 14.02 | 14.14 |
| 26 | WH 542 | 46.63 | 49.75 | 0.51 | 0.49 | 49.18 | 55.75 | 0.56 | 0.57 | 44.25 | 49.25 | 0.54 | 0.53 | 75.55 | 74.35 | 84.64 | 116.68 | 0.47 | 0.55 | 34.99 | 27.84 | 16.37 | 15.17 |
| 27 | WH 1021 | 46.20 | 48.50 | 0.54 | 0.48 | 47.33 | 53.73 | 0.58 | 0.54 | 44.43 | 47.62 | 0.54 | 0.51 | 76.63 | 76.97 | 84.41 | 96.02 | 0.47 | 0.45 | 26.99 | 27.93 | 12.61 | 12.58 |
| 28 | WH 1022 | 49.33 | 56.00 | 0.55 | 0.56 | 51.83 | 62.00 | 0.59 | 0.61 | 49.23 | 55.00 | 0.57 | 0.58 | 77.60 | 77.49 | 103.52 | 130.93 | 0.58 | 0.62 | 29.42 | 27.84 | 16.85 | 17.10 |
| 29 | UAS BW-13354 | 51.35 | 51.65 | 0.55 | 0.57 | 52.25 | 57.65 | 0.61 | 0.62 | 51.58 | 51.65 | 0.54 | 0.59 | 76.57 | 77.08 | 103.18 | 111.98 | 0.57 | 0.53 | 33.10 | 34.61 | 18.90 | 18.19 |
| 30 | UAS BW-13355 | 51.65 | 52.80 | 0.55 | 0.55 | 54.15 | 58.80 | 0.59 | 0.60 | 51.68 | 52.30 | 0.58 | 0.58 | 76.99 | 77.73 | 92.46 | 118.72 | 0.51 | 0.56 | 31.47 | 32.90 | 16.09 | 18.32 |
| 31 | UAS BW-13356 | 55.93 | 59.65 | 0.61 | 0.60 | 56.45 | 67.15 | 0.66 | 0.68 | 56.00 | 57.83 | 0.62 | 0.65 | 73.49 | 71.15 | 110.74 | 125.59 | 0.62 | 0.59 | 35.15 | 32.27 | 21.54 | 18.95 |
| 32 | UAS BW-13357 | 50.80 | 56.08 | 0.56 | 0.57 | 52.65 | 62.08 | 0.61 | 0.62 | 51.78 | 54.83 | 0.57 | 0.58 | 71.40 | 70.75 | 108.91 | 123.69 | 0.61 | 0.58 | 30.61 | 30.86 | 18.47 | 17.86 |
| 33 | UAS BW-13358 | 52.88 | 58.55 | 0.52 | 0.58 | 54.93 | 66.23 | 0.54 | 0.68 | 52.45 | 56.64 | 0.52 | 0.63 | 72.73 | 70.92 | 112.07 | 114.70 | 0.62 | 0.54 | 34.02 | 35.17 | 21.11 | 18.83 |
| 34 | UAS BW-13359 | 49.93 | 55.30 | 0.55 | 0.56 | 51.93 | 63.13 | 0.56 | 0.63 | 51.60 | 54.48 | 0.55 | 0.60 | 78.60 | 76.85 | 104.50 | 117.46 | 0.58 | 0.55 | 32.15 | 33.40 | 18.59 | 16.63 |
| 35 | UAS BW-13360 | 50.58 | 55.98 | 0.55 | 0.51 | 52.40 | 61.73 | 0.58 | 0.57 | 51.18 | 54.60 | 0.57 | 0.54 | 74.00 | 72.81 | 100.22 | 113.17 | 0.56 | 0.54 | 29.29 | 27.84 | 16.25 | 14.78 |
| 36 | UAS BW-13361 | 45.28 | 45.70 | 0.53 | 0.52 | 47.40 | 51.70 | 0.54 | 0.59 | 46.33 | 45.70 | 0.54 | 0.56 | 70.33 | 74.21 | 91.39 | 125.86 | 0.51 | 0.59 | 31.83 | 26.67 | 16.11 | 15.71 |
| 37 | UAS BW-13362 | 47.80 | 54.55 | 0.57 | 0.51 | 49.08 | 60.55 | 0.59 | 0.57 | 48.95 | 52.90 | 0.55 | 0.54 | 78.36 | 77.35 | 82.34 | 115.01 | 0.46 | 0.54 | 26.98 | 22.67 | 12.28 | 12.22 |
| 38 | UAS BW-13363 | 44.25 | 50.23 | 0.54 | 0.51 | 44.93 | 56.23 | 0.56 | 0.62 | 46.63 | 49.98 | 0.56 | 0.56 | 75.86 | 74.40 | 87.43 | 100.78 | 0.49 | 0.47 | 31.68 | 28.76 | 15.32 | 13.61 |
| 39 | UAS BW-13364 | 46.88 | 53.88 | 0.53 | 0.51 | 48.18 | 59.38 | 0.55 | 0.56 | 42.50 | 52.50 | 0.54 | 0.53 | 77.99 | 69.28 | 93.13 | 110.95 | 0.52 | 0.52 | 34.38 | 31.28 | 17.71 | 16.17 |
| 40 | UAS BW-13365 | 46.20 | 48.95 | 0.47 | 0.56 | 48.38 | 54.95 | 0.50 | 0.62 | 45.55 | 49.70 | 0.48 | 0.58 | 74.65 | 73.34 | 88.25 | 112.92 | 0.50 | 0.53 | 29.06 | 25.66 | 14.31 | 13.58 |
| 41 | UAS BW-13366 | 46.38 | 52.43 | 0.51 | 0.53 | 47.50 | 58.43 | 0.60 | 0.59 | 46.55 | 51.68 | 0.54 | 0.56 | 76.64 | 74.25 | 84.39 | 122.60 | 0.47 | 0.58 | 33.40 | 27.22 | 16.33 | 15.61 |
| 42 | UAS BW-13367 | 48.55 | 50.08 | 0.54 | 0.55 | 51.08 | 56.08 | 0.54 | 0.61 | 49.93 | 49.58 | 0.55 | 0.58 | 75.15 | 67.91 | 95.08 | 116.01 | 0.53 | 0.55 | 32.68 | 28.21 | 17.20 | 15.23 |
| 43 | UASBW 12758 | 44.98 | 52.28 | 0.5 | 0.54 | 46.25 | 56.03 | 0.53 | 0.60 | 43.85 | 50.65 | 0.53 | 0.56 | 76.85 | 71.28 | 87.35 | 126.14 | 0.49 | 0.59 | 30.22 | 24.77 | 14.64 | 14.67 |
| 44 | UASBW-10227 | 45.88 | 51.00 | 0.57 | 0.51 | 47.45 | 56.00 | 0.54 | 0.56 | 45.65 | 50.00 | 0.58 | 0.53 | 77.70 | 66.09 | 97.38 | 115.59 | 0.54 | 0.54 | 29.32 | 25.18 | 15.80 | 13.65 |
| 45 | UASBW10453 | 48.35 | 48.68 | 0.51 | 0.52 | 49.58 | 54.68 | 0.55 | 0.59 | 49.50 | 47.93 | 0.54 | 0.55 | 76.93 | 75.69 | 96.51 | 122.56 | 0.54 | 0.58 | 31.57 | 29.35 | 16.85 | 16.64 |
| 46 | UASBW-11948 | 46.88 | 50.40 | 0.53 | 0.56 | 47.60 | 57.08 | 0.59 | 0.62 | 48.48 | 50.08 | 0.56 | 0.58 | 76.52 | 69.45 | 92.78 | 134.78 | 0.51 | 0.63 | 31.94 | 25.67 | 16.42 | 16.13 |
| 47 | UASBW-12380 | 45.70 | 48.68 | 0.56 | 0.55 | 47.33 | 54.75 | 0.55 | 0.62 | 46.38 | 47.97 | 0.55 | 0.56 | 76.24 | 75.71 | 100.19 | 126.79 | 0.56 | 0.60 | 30.89 | 27.04 | 17.13 | 16.06 |
| 48 | UASBW-12876 | 47.65 | 48.20 | 0.51 | 0.51 | 49.80 | 54.20 | 0.54 | 0.56 | 47.33 | 47.70 | 0.54 | 0.53 | 74.61 | 78.64 | 93.93 | 101.61 | 0.52 | 0.48 | 31.72 | 32.54 | 16.48 | 15.47 |
| 49 | UASBW-12877 | 47.13 | 51.38 | 0.52 | 0.53 | 49.50 | 57.38 | 0.57 | 0.59 | 48.03 | 50.88 | 0.55 | 0.56 | 72.60 | 77.38 | 98.40 | 113.42 | 0.55 | 0.54 | 29.03 | 28.75 | 15.80 | 15.28 |
| 50 | UASBW-12878 | 46.78 | 49.55 | 0.54 | 0.54 | 47.43 | 55.55 | 0.58 | 0.60 | 46.50 | 49.05 | 0.55 | 0.57 | 74.91 | 75.03 | 95.56 | 122.42 | 0.53 | 0.58 | 33.36 | 27.13 | 17.62 | 15.54 |

**Where,**

**DH:** Days to Heading, DM: Days to maturity, PH: Plant height (cm), NPT: Number of productive tiller per meter, SL: Spike length (cm), AL: Awn length (cm), SPS: spikelet’s per spike, GPS: grains per spike, TSW: Thousand seed weight (g), GY: Grain yield (q ha^-1^), BMY: Biomass yield (q ha^-1^), HI: Harvest index (%) , GPC: Grain protein content (%), CC-1: Chlorophyll content at booting stage, NDVI -1: NDVI at booting stage, CC-2: Chlorophyll content at anthesis stage, NDVI- 2: NDVI at anthesis stage, CC-3 : Chlorophyll content at grain filling stage, NDVI -3: NDVI at grain filling stage, NHI: Nitrogen harvest index, TNUp: Total nitrogen uptake (kg N ha^-1^), NUpE: Nitrogen uptake efficiency (kg N kg^-1^N), NUtE: Nitrogen utilization efficiency (kg grain kg^-1^N) and NUE: Nitrogen use efficiency (kg grain kg^-1^N).
