# Supplementary material for: Nitrogen use efficiency in bread wheat: Genetic variation and prospects for improvement
Source: PLoS One. 2024 Apr 10;19(4):e0294755. doi: 10.1371/journal.pone.0294755 (PMC11006162; doi:10.1371/journal.pone.0294755)

PLOS ONE

Nitrogen Use Efficiency in Bread Wheat: Genetic Variation and Prospects for Improvement

--Manuscript Draft--

**Manuscript Number:**	PONE-D-23-04727
**Article Type:**	Research Article
**Full Title:**	Nitrogen Use Efficiency in Bread Wheat: Genetic Variation and Prospects for Improvement
**Short Title:**	Nitrogen Use Efficiency in Bread Wheat: Genetic Variation and Prospects for Improvement
**Corresponding Author:**	S A Desai, Postdoc<br>University of Agricultural Sciences Dharwad<br>Dharwad, Karnataka INDIA
**Keywords:**	Bread wheat, nitrogen use efficiency, genotypic variations, phenotyping, nitrogen insensitivity
**Abstract:**	Nitrogen (N) is one of the primary macronutrients required for crop growth and yield. This nutrient is especially limiting wheat yields in the dry and low fertile agro-ecologies having low N in the root zone soil strata. Moreover, majority of farmers in India and South Asia are small to marginal with meagre capacity to invest in costly nitrogen fertilizers. Therefore, there is an immense need to identify lines that use nitrogen efficiently. A set of 50 diverse wheat genotypes consisting of indigenous germplasm lines (05), cultivars released for commercial cultivation (,23) and selected elite lines from CIMMYT nurseries (22) were evaluated in an alpha-lattice design with two replications, a six-rowed plot of 2.5m length for 24 agro morphological, physiological and NUE related traits during two consecutive crop seasons in an N-depleted precision field under two different N levels of 50%-N50 (T1) and 100%-N100 (T2) of recommended N, i.e., 100 kg/ha. Analysis of variance revealed significant genetic variation among genotypes for all the traits studied. About 11.36% yield reduction was observed at reduced N levels. Significant correlations among NUE traits and yield component traits were observed which indicated pivotal role of N remobilization to the grain in enhancing yield levels. Among N-insensitive genotypes identified based on their yielding ability at low N levels, UASBW13356, UASBW13358, UASBW13354, UASBW13357 and KRL1-4 showed their inherent genotypic plasticity toward N application. The genotypes with more yield and high to moderate NUtE can be used as parents for the breeding of N efficient genotypes for marginal agro-ecologies. Low N tolerant genotypes identified from the current investigation may be further utilized in the identification of genomic regions responsible for NUE and its deployment in wheat breeding programs. The comprehensive data of 24 traits under different nitrogen levels for diverse genotypes from India and global sources (mainly CIMMYT) should be useful for supporting breeding for NUE and thus will be of great help for small and marginal farmers in India and South Asia.
**Order of Authors:**	Suma S Biradar, Ph.D
	Mahalaxmi K Patil, Ph.D in Genetics and Plant Breeding
	S A Desai, Postdoc
	Sanjay Kumar Singh, Ph.D
	Rudra Naik V, Ph.D
	Kumar Lamani, Ph.D
	Arun K Joshi, Post doc
**Additional Information:**	
**Question**	**Response**
**Financial Disclosure**	The author(s) received no specific funding for this work.

Powered by Editorial Manager® and ProduXion Manager® from Aries Systems Corporation

Enter a financial disclosure statement that describes the sources of funding for the work included in this submission. Review the submission guidelines for detailed requirements. View published research articles from *PLOS ONE* for specific examples.<br><br>This statement is required for submission and **will appear in the published article** if the submission is accepted. Please make sure it is accurate.<br><br>**Unfunded studies**<br>Enter: *The author(s) received no specific funding for this work.*<br><br>**Funded studies**<br>Enter a statement with the following details:<br>• Initials of the authors who received each award<br>• Grant numbers awarded to each author<br>• The full name of each funder<br>• URL of each funder website<br>• Did the sponsors or funders play any role in the study design, data collection and analysis, decision to publish, or preparation of the manuscript?<br>• **NO** - Include this sentence at the end of your statement: *The funders had no role in study design, data collection and analysis, decision to publish, or preparation of the manuscript.*<br>• **YES** - Specify the role(s) played.<br><br>* typeset	
**Competing Interests**<br><br>Use the instructions below to enter a competing interest statement for this submission. On behalf of all authors, disclose any competing interests that could be perceived to bias this work—acknowledging all financial support and any other relevant financial or non-financial competing interests.<br><br>This statement is required for submission and **will appear in the published article** if	The authors have declared that no competing interests exist.

the submission is accepted. Please make sure it is accurate and that any funding sources listed in your Funding Information later in the submission form are also declared in your Financial Disclosure statement.<br><br>View published research articles from *PLOS ONE* for specific examples.<br><br>**NO authors have competing interests**<br><br>Enter: *The authors have declared that no competing interests exist.*<br><br>**Authors with competing interests**<br><br>Enter competing interest details beginning with this statement:<br><br>*I have read the journal's policy and the authors of this manuscript have the following competing interests: [insert competing interests here]*<br><br>* typeset	
**Ethics Statement**<br><br>Enter an ethics statement for this submission. This statement is required if the study involved:<br><br>• Human participants<br>• Human specimens or tissue<br>• Vertebrate animals or cephalopods<br>• Vertebrate embryos or tissues<br>• Field research<br><br>Write "N/A" if the submission does not require an ethics statement.<br><br>General guidance is provided below. Consult the submission guidelines for detailed instructions. **Make sure that all information entered here is included in the Methods section of the manuscript.**	N/A

Powered by Editorial Manager® and ProduXion Manager® from Aries Systems Corporation

**Format for specific study types**<br><br>**Human Subject Research (involving human participants and/or tissue)**<br>• Give the name of the institutional review board or ethics committee that approved the study<br>• Include the approval number and/or a statement indicating approval of this research<br>• Indicate the form of consent obtained (written/oral) or the reason that consent was not obtained (e.g. the data were analyzed anonymously)<br><br>**Animal Research (involving vertebrate animals, embryos or tissues)**<br>• Provide the name of the Institutional Animal Care and Use Committee (IACUC) or other relevant ethics board that reviewed the study protocol, and indicate whether they approved this research or granted a formal waiver of ethical approval<br>• Include an approval number if one was obtained<br>• If the study involved *non-human primates*, add *additional details* about animal welfare and steps taken to ameliorate suffering<br>• If anesthesia, euthanasia, or any kind of animal sacrifice is part of the study, include briefly which substances and/or methods were applied<br><br>**Field Research**<br><br>Include the following details if this study involves the collection of plant, animal, or other materials from a natural setting:<br>• Field permit number<br>• Name of the institution or relevant body that granted permission	
**Data Availability**<br><br>Authors are required to make all data underlying the findings described fully available, without restriction, and from the time of publication. PLOS allows rare exceptions to address legal and ethical concerns. See the PLOS Data Policy and FAQ for detailed information.	No - some restrictions will apply

Powered by Editorial Manager® and ProduXion Manager® from Aries Systems Corporation

A Data Availability Statement describing where the data can be found is required at submission. Your answers to this question constitute the Data Availability Statement and **will be published in the article**, if accepted.<br><br>**Important:** Stating 'data available on request from the author' is not sufficient. If your data are only available upon request, select 'No' for the first question and explain your exceptional situation in the text box.<br><br>Do the authors confirm that all data underlying the findings described in their manuscript are fully available without restriction?	
**Describe where the data may be found in full sentences. If you are copying our sample text, replace any instances of XXX with the appropriate details.**<br><br>• If the data are **held or will be held in a public repository**, include URLs, accession numbers or DOIs. If this information will only be available after acceptance, indicate this by ticking the box below. For example: *All XXX files are available from the XXX database (accession number(s) XXX, XXX.).*<br>• If the data are all contained **within the manuscript and/or Supporting Information files**, enter the following: *All relevant data are within the manuscript and its Supporting Information files.*<br>• If neither of these applies but you are able to provide **details of access elsewhere**, with or without limitations, please do so. For example:<br><br>*Data cannot be shared publicly because of [XXX]. Data are available from the XXX Institutional Data Access / Ethics Committee (contact via XXX) for researchers who meet the criteria for access to confidential data.*<br><br>*The data underlying the results presented in the study are available from (include the name of the third party*	All relevant data are within the manuscript

and contact information or URL).<br>• This text is appropriate if the data are owned by a third party and authors do not have permission to share the data.<br><br>* typeset	
Additional data availability information:	

Powered by Editorial Manager® and ProduXion Manager® from Aries Systems Corporation

Manuscript

Click here to access/download;Manuscript;Nitrogen use efficiency in Bread wheat_PLoS ONE_09-03-2023.doc

Nitrogen Use Efficiency in Bread Wheat: Genetic Variation and Prospects for Improvement

Suma S Biradar[1], Mahalaxmi K Patil[2], SA Desai[2*], Sanjay K Singh[3*], V Rudra Naik[4], Kumar Lamani[5] and Arun K Joshi[6]

[1]AICRP on Wheat, MARS, University of Agricultural Sciences, Dharwad 580 005, India.

[2] Department of Genetics & Plant Breeding, University of Agricultural Sciences, Dharwad 580 005, India.

[3] ICAR-Indian Agricultural Research Institute, New Delhi-110012, India

[4]Directorate of Research, University of Agricultural Sciences, Dharwad 580 005, India.

[5]Department of Agronomy, University of Agricultural Sciences, Dharwad 580 005, India

[6]CIMMYT- India, NASC Complex, DPS Marg, New Delhi-110012, India

***Corresponding authors**:

SA Desai, desaisa@uasd.in

Sanjay K Singh, Sanjay.Singh4@icar.gov.in

Abstract:

Nitrogen (N) is one of the primary macronutrients required for crop growth and yield. This nutrient is especially limiting wheat yields in the dry and low fertile agro-ecologies having low N in the root zone soil strata. Moreover, majority of farmers in India and South Asia are small to marginal with meagre capacity to invest in costly nitrogen fertilizers. Therefore, there is an immense need to identify lines that use nitrogen efficiently. A set of 50 diverse wheat genotypes consisting of indigenous germplasm lines (05), cultivars released for commercial cultivation (,23) and selected elite lines from CIMMYT nurseries (22) were evaluated in an alpha-lattice design with two replications, a six-rowed plot of 2.5m length for 24 agro morphological, physiological and NUE related traits during two consecutive crop seasons in an N-depleted precision field under two different N levels of 50%-N50 (T1) and 100%-N100 (T2) of recommended N, i.e., 100 kg/ha. Analysis of variance revealed significant genetic variation among genotypes for all the traits studied. About 11.36% yield reduction was observed at reduced N levels. Significant correlations among NUE traits and yield component traits were observed which indicated pivotal role of N remobilization to the grain in enhancing yield levels. Among N-insensitive genotypes identified based on their yielding ability at low N levels, UASBW13356, UASBW13358, UASBW13354, UASBW13357 and KRL1-4 showed their inherent genotypic plasticity toward N application. The genotypes with more yield and high to moderate NUtE can be used as parents for the breeding of N efficient genotypes for marginal agro-ecologies. Low N tolerant genotypes identified from the current investigation may be further utilized in the identification of genomic regions responsible for NUE and its deployment in wheat breeding programs. The comprehensive data of 24 traits under different nitrogen levels for diverse genotypes from India and global sources (mainly CIMMYT) should be useful for supporting breeding for NUE and thus will be of great help for small and marginal farmers in India and South Asia.

Keywords: Bread wheat, nitrogen use efficiency, genotypic variations, phenotyping, nitrogen insensitivity.

1. Introduction

Nitrogen (N) is an essential nutrient for optimizing plant growth and reproduction and therefore, applying N fertilizer is an essential practice to secure productivity in diverse cropping systems. Plants take N from both atmospheric air and soil minerals, but crop plants are inefficient in the acquisition and utilization of applied nitrogen. The efficient use

of N by plants is determined by their ability to absorb naturally available N or applied N fertilizers. Current global nitrogen (N) demand stands at about 110.19 million metric tons with a projected annual increase of ~1.3% in the future (FAO, 2019) which equates to global N fertilizer consumption of more than 110 Mt per annum of which half of the total is being used for the production of major cereal crops i.e., maize, rice, and wheat (Ladha *et al*., 2016). Indian agriculture consumes over 17 million tons of N fertilizer per year. Studies have shown that more than 50% of applied N fertilizers are unused by crops (Raun and Johnson, 1999; Lassaletta *et al*., 2014) and this unabsorbed N fertilizer in the soil is lost to the environment by leaching, denitrification, runoff, and atmospheric release through volatilization and becomes a major environmental concern (Vitousek *et al*., 1997; Zafar and Muhammad, 2007). Surplus nitrogen also pollutes freshwater streams and air which is hazardous to most living species (Hickman *et al*., 2014; Russo *et al*., 2017). Besides, excess usage of N fertilizer not only decreases the efficiency of nutrient use but also affects the rate of economic returns per unit of chemical fertilizer applied. The effect of negative environmental and economic impacts could be reduced through better agronomic practices and also by utilizing N efficient lines with improved nitrogen use efficiency (NUE) (Raun *et al*., 1999). Hence, the development of crop plants with more N-use efficiency (NUE) with efficient nitrogen usage is, therefore, an important research goal in enhancing future food production capabilities to achieve greater agricultural sustainability (Hirel *et al*., 2007).

Wheat (*Triticum aestivum* L.) is one of the staple food crops that ensures food and nutritional security for the world's poorest people living in different agro-ecological zones. In the Indian agriculture scenario, wheat is grown under diverse production conditions including low fertile and rainfall dependent central and peninsular areas often facing drought during different stages of crop growth. In such areas, farmers apply a high dose of N fertilizer for maximizing grain yield (GY), but excessive usage of N fertilizer leads to low NUE of the crop along with various environmental hazards. Further many farmers lack knowledge about the ideal dose of fertilizer to harvest the real yield potential. The majority (90%) of such farmers are small and marginal and do not have capacity to take risk of applying high inputs. Hence, yield improvement under low nitrogen input would be extremely beneficial for economic and environmentally sustainable cultivation of wheat crop.

Similar to grain yield, nitrogen use efficiency (NUE) is also a complex trait, which is associated with various morphological, physiological, molecular, and biochemical changes in plants throughout the life cycle. For a clear understanding of this complex nature, studies of various physiological traits and their close correlation with one or more economically important traits like GY are foremost critical. Nevertheless, it will help in selecting low N tolerant/high yielding lines at different N conditions (Monostori *et al*., 2016). In general, plant function is always associated with chlorophyll content, which directly indicates the N status of the leaf (Yang *et al*., 2014). Leaf chlorophyll content and photosynthetic capacity are appropriate benchmarks for identifying high NUE (HNUE) genotypes under low N conditions during field trials (Vijayalakshmi *et al*., 2015; Kiran *et al*., 2016). Leaf N status was usually measured by using a hand-held optical chlorophyll meter to monitor the leaf nitrogen status and chlorophyll content.

NUE has several definitions based on agronomic, genetic, or physiological studies (Fageria *et al*., 2008). NUE is the efficiency of nitrogen recovery from applied fertilizer, or the N available to the crop, and this gives rise to the 33% efficiency of crop recovery (Raun and Johnson, 1999; Zhang *et al*., 2015). Alternatively, it is often considered a productivity index and defined as the yield produced per unit of available N to the crop expressed in terms of kg yield per kg of available N (Moll *et al*., 1982; Barraclough *et al*., 2010). NUE

included two components namely, N uptake efficiency (NUpE) and N utilization efficiency (NUtE) and is mathematically the product of NUpE x NUtE (Good *et al*., 2004; Hakeem *et al*., 2012; Vijayalakshmi *et al*., 2013). NUpE is the ability of the plant to take up N from the soil. It is estimated as the ratio of N taken up by the plant per unit of N available from the soil and applied fertilizer and is expressed as kg N (in the crop) per kg N (available). NUtE is the ability to use N to produce gain yield which is estimated as the grain yield produced per unit of N taken up by the plant and is also expressed as kg (grain) per kg N (Ladha *et al*.,1998; Hirel *et al*., 2007).
Studies on genotypic variations under low and recommended N for NUE traits in various crops spurred breeding activities to develop N use-efficient genotypes possessing high yield sustainability under low N conditions (Borrell *et al*., 1998; Muchow, 1998; Inthapanya *et al*., 2000; Le Gouis *et al*., 2000; Presterl *et al*., 2003; Hirel *et al*., 2007; Anbessa *et al*., 2009; Namai *et al*., 2009; Le Gouis *et al*., 2010; Vijayalakshmi *et al*., 2015). There have been continuous improvements in N use efficiency (NUE) of crops over the years along with increases in crop yield (Ortiz-Monasterio *et al*., 1997; Ciampitti and Vyn, 2012; Sadras and Lawson, 2013; Cormier et al., 2016) and further extension of the genetic pool through introgression of landraces and ancestral germplasm is expected to support breeding programs in driving future NUE-based outcomes (Hawkesford, 2014; Cormier et al., 2016). A study identified 333 genomic regions associated to 28 traits related to NUE in winter wheat (Cormier *et al*., 2014). Cormier *et al.* (2016) has reviewed the molecular studies done for NUE. However, However, NUE and its related traits are polygenic and influenced by genetic background.

The high NUE/ N-insensitive genotypes (NIS-top grain yielders) give more or equal GY with minimal application of N fertilizer compared to recommended N fertilizer conditions (Hawkesford, 2017). The understanding of GY and its associated NUE traits performance is still lagging in wheat but few studies had explored genotypic variations for NUE at different N levels. A significant genetic variability was observed for NUtE than NUpE at low N inputs in wheat by Gaju *et al*., (2011). However, Maman *et al*., (2006) concluded that the NUtE trait was less responsive than the N uptake with increased N levels. In broader perspectives, NUE is an output of available N uptake, its efficient utilization, and ultimately remobilization to grain. Several studies in cereals suggested enhanced NUE through improved NUtE under low N conditions (Moll *et al*., 1982; Good *et al*., 2004). The nitrogen harvest index (NHI) is another useful measure of the efficiency of N use, which is the fraction of total N taken up by the crop which is partitioned to the grain. NHI is independent of yield and uptake efficiency, and a low yielding crop may have a high NHI (Hawkesford and Riche, 2020).

The green revolution period in India focused primarily on the responsiveness of wheat genotypes to high-input supply. In the present scenario of climate change, realizing consistent output (yield and quality) of the genotype under varying levels of nitrogen input is a major challenge to wheat improvement programmes. Identifying promising genotypes for enhanced NUE in wheat and estimating genetic variability can provide useful guidance to breeders (Gaju *et al*., 2011). Keeping high yield when N supply is reduced and/or increasing yield when N supply is continuous may be two strategies for NUE enhancement. Nitrogen production costs, environmental pollution owing to nitrate leaching (Pathak *et al*., 2008), and greenhouse gas volatilization necessitate improvement of wheat nitrogen use efficiency at a reduced N supply. Simultaneously, GY is a complex trait controlled by a network of multiple traits and their associations. Hence, uncovering the genetic basis of GY, and other related NUE traits under low and high N conditions is a prerequisite to understanding the mechanism and also identifying ideal NUE associated traits for selection. Nitrogen use

efficient crop varieties can be a great choice for ensuring sustainability in farming systems and meeting future customer requirements, particularly in the face of changing environment caused by climate change. Thus, the current study aimed to evaluate a diverse set of wheat genotypes for their response to GY and NUE related traits under different N levels and also to determine the suitable NUE traits for selection. This study provides useful information for uncovering the physiological and genetic basis of GY and its related traits under low N which further facilitates the development of low N stress resilient wheat cultivars.

2. Material and Methods

2.1 Selection of genotypes

The genotypes included in the study consisted of fifty bread wheat genotypes representing high, medium, and low NUE based on multi-location and multi-year evaluation. These genotypes represent indigenous elite lines (DTW2011-56, GW2013-540, MP1293, RAJ4248, UAS323), cultivars released for commercial cultivation (DBW14, DL153-2, GW322, HD2189, HD2733, HD2967, HI1500, HI1530, HPW251, K9107, Kalyansona, KRL1-4, KRL237, MP4010, NP846, PBW175, PBW343, RAJ1972, UAS304, WH147, WH542, WH1021, WH1022) and selected elite lines from CIMMYT trials/nurseries (UASBW10227, UASBW10453, UASBW11948, UASBW12380, UASBW12758, UASBW12876, UASBW12877, UASBW12878, UASBW13354, UASBW13355, UASBW13356, UASBW13357, UASBW13358, UASBW13359, UASBW13360, UASBW13361, UASBW13362, UASBW13363, UASBW13364, UASBW13365, UASBW13366, UASBW13367). The investigation was carried out for two years during *rabi* 2016-17 and 2017-18 at All India Coordinated Wheat Improvement Project, Main Agricultural Research Station, University of Agricultural Sciences, Dharwad located at 15.48^{o}N latitude and 74.98^{o}E longitude and falls in agro-climatic Zone 8 of Karnataka state in Peninsular India.

2.2 Experimental design, treatment, and crop management

The experiment was laid out in an Alpha-lattice design at two treatment levels of nitrogen (T_1 and T_2) with two replications. The T_1 consisted of soil N + 50% of the recommended nitrogen dose (RND) of 100 kg N ha^{-1} which is equivalent to 50kg/ha^{-1} whereas T_2 consisted of soil N +RND of 100 kg N ha^{-1}. The genotypes were randomly allotted to plots in each treatment in each replication and grown in 6 row plots of 2.5 meter length with 20 cm row spacing. The nitrogen fertilizer dose was calculated as per the treatments and was applied to each plot. The soil samples from the experimental sites were collected from 0 to 30 cm soil depth before the start of the field experiment and after harvest of the crop and N was analyzed using standard procedure. All other recommended agronomic package of practices was adopted to raise a good crop.

2.3 Observations

Data on 24 agro-morphological, physiological, and NUE related traits were recorded from phase 41 to phase 92 of the Zadoks scale (Zadoks *et al*., 1974) for wheat plants in the four central rows of each plot. The agro-morphological traits included grain yield (q ha-1), agro-morphological traits namely, days to heading, days to flowering, plant height (cm), number of productive tiller per meter row length, spike length (cm), awn length (cm), spikelet number per spike, grains number per spike, 1000-grains weight (g), biomass yield (q ha-1) and harvest index (%); Physiological traits namely, chlorophyll content (SPAD) and normalized difference vegetation index (NDVI) were recorded at booting (SPAD 1, NDVI-1), anthesis (SPAD-2, NDVI-2) and grain filling (SPAD-3, NDVI-3) stages. The chlorophyll

content was measured by a Chlorophyll meter or Spad-meter and NDVI was measured by the Trimble GreenSeeker handheld crop sensor using standard procedures. Nitrogen use efficiency (NUE) and its related parameters like nitrogen uptake efficiency (NUpE), nitrogen utilization efficiency (NUtE), nitrogen harvest index (NHI), and total nitrogen uptake (TNUp) were worked out according to Moll *et al*., (1982) and Ayadi *et al*., (2012). NHI was calculated as [Grain N/ (Grain N + straw N)] *100 and expressed in percentage. The grain N (%) and straw N (%) contents were analyzed by the Micro-Kjeldahl digestion method as per the FAO guide to laboratory establishment for plant nutrient analysis (FAO, 2008). TNUp (kg N ha-1) was determined as the sum of nitrogen in straw and grain at harvest. NUpE was calculated as total N uptake/crop N supply where crop N supply includes fertilizer N + soil mineral N at planting. NUtE was calculated as grain yield/total N uptake. NUE was measured as Grain yield/ total nitrogen supply from the soil and applied fertilizer. The values for NUE, NUtE and NUpE were expressed in kg kg^{-1}.

2.4 Statistical analysis

Before pooling the data across environments, the ANOVA assumption was tested for its homogeneity using the Bartlet test (Snedecor and Cochran, 1989). The ratio of the highest error means square and the smallest error mean square value was compared with the F table and it showed non-significant results for all the traits hence, two years of data across environments were pooled. From pooled data, the analysis of variance (ANOVA) was done as per Panse and Sukhatme (1967). Critical difference was estimated to see significant differences among the genotypes for various studied traits. The genotypic and phenotypic coefficients of variation [Burton 1952] and heritability (Lush, 1940; Hanson *et al*., 1956) were worked out and the traits were categorized as low, medium and high based on genetic parameters (Shivasubramanian and Menon, 1973; Robinson *et al*., 1949;). The correlation among all the traits studied under both the nitrogen levels was worked out using Karl Pearson's simple correlation coefficient method.

For understanding the effect of nitrogen doses on different genotypes concerning yield as well as NUE, various indexes namely, stress susceptibility index (Fischer and Maurer, 1978), percentage reduction (Chouken *et al*., 2006) and yield stability index (Bouslama and Schapaugh, 1984) were also estimated to identify promising genotypes. As grain yield and NUE measurement are reflective of each other in totality, grain yield was used to estimate these indices.

2. Results

3.1Trait specific variability among genotypes

The computed mean sum of squares for various traits namely, chlorophyll content at booting stage (CC-1), anthesis stage (CC-2), grain filling stage (CC-3), NDVI at booting stage (NDVI-1), anthesis stage (NDVI-2), grain filling stage (NDVI-3), days to heading (DH), days to maturity (DM), plant height (PH), number of productive tiller per meter row length (TPM), spike length (SL), awn length (AL), number of spikelet's per spike (SPS), number of grains per spike (GPS), thousand grains weight (TGW), biomass yield (BMY), harvest index (HI), grain protein content (GPC), nitrogen harvest index (NHI), total nitrogen uptake (TNUp), nitrogen uptake efficiency (NUpE), nitrogen utilization efficiency (NUtE) and nitrogen use efficiency (NUE) showed that genotypes differed significantly for all the traits which indicated the existence of variation among genotypes for various traits studied. A wide range was observed for all the traits evaluated under T1 (soil N + 50 kg Nha^{-1}) and T2 (Soil N + 100 kg Nha^{-1}) as shown in Table.1. In general, the mean values for all the traits

except HI, NHI, NUtE and NUE were higher at full/recommended fertilizer dose as compared to reduced nitrogen dose. A similar trend was also observed for trait specific maximum values where all the traits except NDVI-1, SL, HI and NUE showed higher values under T2 as compared to T1.

3.1.1. Yield and agro-morphological traits

The present investigation included 12 agro morphological traits including grain yield. A wide range was observed for these yield and agro-morphological traits under both the nitrogen levels. In T1 (N50), the average GY of the tested genotypes was 28.65 q/ha and ranged from 16.87 to 38.91q/ha whereas in T2 (N100), GY ranged from 20.40 q/ha to 40.36q/ha with a mean of 32.32 q/ha. Nitrogen in limited condition (N50) resulted in 11.36% reduction in GY compared to N100. A higher mean BMY of 105.9 q/ha with a range of 79.1 to 132.4q/ha was recorded in T2 as compared to 90.8 q/ha mean BMY with range of 73.0 to 108.7q/ha under the T1 condition that showed 14.25% reduction. Interestingly, 1.3% lower HI was observed in T2 (31.5%) as compared to T1 (31.9%) condition. TPM, GPS and TGW are major yield component traits that significantly contribute to higher yield realization. The results showed a higher mean of 97.3 TPM, 50.1 GPS and 37.0g TGW in the T2 condition as compared to 86.0 TPM, 47.0 GPS and 34.1g TGW in the T1 condition indicating 11.7, 6.19, and 7.81 percent.

Table 1:Pooled ANOVA and genetic parameters for morpho-physiological, yield yield attributes, NUE and NUE related traits in 50 genotypes of bread wheat under T_1 (soil N + 50 kg Nha-1) and T2 (Soil N + 100 kg Nha-1)															
**Traits**	**F statistics (Genotypes at 49 df)**		**Mean**		**Range**		**PCV**		**GCV**		**h^2bs (%)**		**Reduction in grain yield at T1 than T2 (%)**	**CD @ 5 % T1**	**CD @ 5 % T2**
	**T1**	**T2**	**T1**	**T2**	**T1**	**T2**	**T1**	**T2**	**T1**	**T2**	**T1**	**T2**			
CC 1	2.54**	5.22**	47.7	51.63	42.95-55.93	45.70-59.65	6.7	6.45	4.42	5.32	43.49	67.82	7.61	4.89	3.84
CC 2	3.70**	6.82**	49.24	57.68	42.00-56.45	51.70-67.15	5.6	6.25	4.25	5.39	57.47	74.43	14.63	3.92	3.66
CC 3	5.46**	5.00**	47.87	50.96	40.50-56.00	45.70-57.83	7.03	6.12	5.84	5.0	69.05	66.71	6.06	3.82	3.62
NDVI 1	4.14**	3.41**	0.54	0.53	0.47-0.61	0.48-0.60	5.1	5.64	3.99	4.17	61.06	54.69	-1.89	0.03	0.04
NDVI 2	3.48**	4.67**	0.57	0.6	0.50-0.66	0.53-0.68	6.33	5.7	4.71	4.58	55.37	64.73	5.00	0.05	0.04
NDVI 3	3.83**	2.86**	0.55	0.56	0.48-0.62	0.51-0.65	5.43	5.49	4.16	3.81	58.61	48.15	1.79	0.04	0.04
DH	14.20**	22.97**	59.42	62.25	50.75-65.25	55.25-68.00	5.52	4.66	5.14	4.46	86.84	91.66	4.55	2.39	1.68
DM	13.93**	31.48**	94.73	99.99	81.75-104.50	91.75-110.50	5.25	3.84	4.88	3.72	86.6	93.84	5.26	3.76	1.92
PH	2.21**	2.28**	74.41	80.02	61.48-90.70	63.98-93.98	9.14	9.88	5.62	6.17	37.77	39.01	7.01	10.89	12.51
TPM	4.50**	2.76**	85.95	97.32	60.00-111.25	79.50-121.50	15.68	11.53	12.51	7.89	63.63	46.76	11.68	16.39	16.46
SL	2.03**	3.03**	7.95	8.3	6.84-10.07	6.92-9.96	10.56	9.3	6.16	6.61	34.05	50.4	4.22	1.44	1.10
AL	2.28**	9.41**	6.27	6.72	5.43-8.29	4.72-8.50	12.48	14.15	7.8	12.72	39.02	80.78	6.70	1.23	0.84
SPS	4.82**	4.37**	16.44	17.8	13.20-19.80	15.25-20.30	9.41	5.91	7.63	4.68	65.65	62.77	7.64	1.83	1.29
GPS	3.68**	4.56**	47	50.1	37.60-57.50	42.75-57.75	9.34	6.09	7.07	4.87	57.3	64.02	6.19	5.98	3.67
TGW	3.71**	11.53**	34.1	36.99	29.13-43.05	31.58-46.40	10.18	8.23	7.72	7.55	57.53	84.03	7.81	4.59	2.46
GY	5.62**	7.09**	28.65	32.32	16.87-38.91	20.40-40.36	16.22	11.4	13.55	9.89	69.79	75.27	11.36	5.18	3.94
BMY	4.29**	4.02**	90.81	105.9	72.95-108.71	79.13-132.38	11.22	15.6	8.85	12.1	62.22	60.13	14.25	12.59	21.51
HI	11.14**	8.08**	31.89	31.49	20.77-52.12	16.88-50.01	20.47	21.61	18.71	19.08	83.53	77.98	-1.27	5.38	6.70
GPC	2.66**	2.74**	12.06	12.56	10.56-13.29	10.85-13.70	7.49	6.81	5.04	4.65	45.35	46.5	3.98	1.34	1.26
NHI	6.84**	2.22**	75.83	73.79	70.03-78.60	66.09-78.64	2.97	4.67	2.57	2.87	74.52	37.81	-2.76	2.29	5.80
TNUp	6.02**	2.57**	94.59	116.24	71.86-113.11	88.97-134.78	10.81	9.1	9.15	6.04	71.54	43.97	18.63	10.96	18.50
NUpE	6.23**	2.57**	0.52	0.54	0.40-0.63	0.42-0.63	14.54	9.67	12.36	6.42	72.34	43.98	3.70	0.06	0.09
NUtE	4.20**	5.35**	30.16	28.08	22.11-35.15	17.95-35.17	10.63	12.24	8.34	10.13	61.54	68.5	-7.41	4.03	4.40
NUE	5.83**	8.97**	15.85	15.16	9.36-21.54	9.57-18.95	16.54	12.02	13.91	10.75	70.73	79.95	-4.55	2.85	1.70

PCV, GCV- Phenotypic and genotypic coefficients of variation, h2bs- heritability broad sense,CC1, CC2, CC3 & NDVI1, NDVI2, NDVI3- Chlorophyll contents and NDVI at booting, anthesis and grain filling stages, respectively, DH- Days to heading, DM- Days to maturity, PH- Plant height, TPM- Number of productive tiller per meter, SL- Spike length, AL-Awn length, SPS-Number of spikelet's per spike, GPS-Number of grains per spike, TGW-Thousand grain weight, BY- Biomass yield, HI- Harvest index, GPC-Grain protein content, NHI- Nitrogen harvest index, TNUp-Total nitrogen uptake, NUpE-Nitrogen uptake efficiency, NUtE-Nitrogen utilization efficiency, NUE-Nitrogen use efficiency

reductions for TPM, GPS and TGW respectively under T1 condition. The lower mean values for other morphological traits DH, DM, PH, SL, AL and SPS were observed in T1 with 4.6, 5.3, 7.0, 4.2, 6.7 and 7.6 percent reduction, respectively as compared to mean values for these traits in the T2 condition.

3.1.2 Physiological parameters

Chlorophyll content and NDVI values are two physiological parameters that directly indicate the phenotypic nitrogen levels in crop plants based on the greenness of leaves. Chlorophyll content (CC-1, CC-2, CC-3) and NDVI values (NDVI-1, NDVI-2, NDVI-3) were measured at booting, anthesis, and grain filling stages, respectively which also showed a wide range in both the conditions. Higher mean values of 51.6, 57.7 and 51.0 chlorophyll content at all three stages were recorded in T2 as compared to 47.7, 49.2 and 47.9 in the T1 condition with a reduction of 7.6%, 14.6% and 6.1%, respectively. Higher chlorophyll content was observed at the anthesis stage in both T1 and T2 conditions. A similar trend was observed for NDVI at anthesis and grain filling stages with 5.0% and 1.8% reduction in the T1 but NDVI at booting stage was a little higher in T1 as compared to T2.

3.1.3 NUE associated traits

Nitrogen based six traits were estimated in the present investigation (Table.1). The NUE in T1 ranged from 9.4 to 21.5 with an average of 15.9 kg grain /kg N whereas it was 15.2 kg grain /kg N with a range of 9.6 to 19.0 in T2 which indicated 4.6% reduction as compared to T1. NUE is mainly dependent on TNUp, NupE and NutE. The results indicated an increase of TNUp with increased application of N fertilizer. It ranged from 71.9 to 113.1 with an average of 94.6 kg N/ha in T1 and decreased by 18.6% as compared with T2 (N100) where it ranged from 89.0 to 134.8 with a mean of 116.2 kg N/ha. Similarly, NupE was 0.52 and 0.54 with a range of 0.40 to 0.63 and 0.42 to 0.63 in T1 and T2, respectively indicating 3.7% reduction in T1. A large variation was observed for NUtE of genotypes which ranged from 22.1 to 35.2 with an average of 30.2 under T1 and 18.0 to 35.2 with a mean of 28.1 in T2. The overall mean showed 7.4% reduction in NUtE in T2 due to poor performance of some of the genotypes which was reflected in lower values of range in T2. The NHI varied from 70.0 to 78.6% with an average of 75.8% in the T1 condition whereas it ranged from 66.1 to 78.6% with a mean of 73.8% in T2 indicating 2.76% reduction as compared to T1. Grain protein content (GPC) is an important trait depending on N uptake and its utilization and in this study, it was approx. 4.0% higher in T2 as compared to the mean of T1. The GPC ranged from 10.9-13.7% with a mean of 12.6% in T2 as compared to 12.1% in T1 with a range of 10.6 to 13.3%.

3.2 Genetic parameters

Genetic parameters namely phenotypic (PCV) and genotypic (GCV) coefficients of variation, heritability in the broad sense (h^2bs) were worked out as shown in Table.1.

3.2.1 Coefficients of Variation

In this study, phenotypic (PCV) and genotypic (GCV) coefficients of variation were estimated in T1 and T2 conditions. In general, higher PCV values were observed as compared to GCV values at both the nitrogen levels. The PCV values ranged from 2.97 (NHI) to 20.47 (HI) under T1 whereas it ranged from 3.84 (DM) to 21.61 (HI) under T2 condition. High PCV of 20.47 and 21.61 was observed only for harvest index under T1 and T2, respectively. The traits TPM (15.68, 11.53), AL (12.48, 14.15), GY (16.22, 11.4), BMY (11.22, 15.6), NUtE (10.63, 12.24) and NUE (16.54, 12.02) have moderate PCV values (10-20) whereas other

traits showed low PCV values (<10) under T1 and T2, respectively. Similarly, GCV ranged from 2.57 and 2.81 for NHI to 18.71 and 21.08 for HI under T1 and T2, respectively. Moderate GCV values for HI (18.71, 19.08) and NUE (13.91, 10.75) were observed under both T1 and T2. Moderate GCV values were also observed for TPM (12.51), GY (13.55), and NUpE (12.36) under T1 and for AL (12.72), BMY (12.1), and NUtE (10.13) under T2. Other traits showed low GCV values in T1 and T2.

3.2.2 Heritability

The heritability values in broad sense ranged from 34.05% for SL to 86.84% for DH in T1 and 37.81% for NHI to 93.84% for DM in T2 (Table.1). At reduced nitrogen level (T1), the highest heritability was observed for DH (86.84) followed by DM (86.60) and HI (83.53) whereas it was highest for DM (93.84) followed by DH (91.66), TGW (84.03), and AL (80.78) at full nitrogen dose (T2). Additionally, high heritability (>60%) was observed for CC-3 (69.1, 66.7), SPS (65.7, 62.8), GY (69.8, 75.3), BMY (62.2, 60.1), NUE (70.7, 80.0) and NUtE (61.5, 68.5) at both T1 and T2, respectively. Traits NHI (74.5), TNUp (71.5) and NUpE (72.3), TPM (63.6) and NDVI-1 (61.1) showed high heritability under T1 only whereas CC-2 (74.4), CC-1 (67.8), NDVI-2 (64.7) and GPS (64.0) showed high heritability under T2 only. Plant height showed low heritability under both T1 (37.8), and T2 (39.0) conditions.

3.3 Phenotypic Correlation

Character association was studied among the genotypes using Karl Pearson's simple correlation coefficient method to identify the interrelation among the traits for both the N levels. Nitrogen use efficiency was measured as grain yield divided by available N soil (soil N + fertilizer N) due to which the correlation of nitrogen use efficiency and grain yield at both levels of nitrogen treatments showed similar results (Fig. 1). Therefore, the correlation values with NUE were estimated as shown in Table.2. The results indicated a positive and highly significant correlated response of nitrogen use efficiency with DM, HI, TGW, GPS, SPS, CC-1, CC-2, CC-3, NDVI-1, NDVI-2, NDVI -3 TNUp, NUpE, NUtE under both T1 and T2 conditions. In addition, similar correlation of NUE with TPM and SL in T1 and with DH and GPC in T2 was also observed. Among these correlations, NUE showed a very strong correlation with HI (0.82), NUpE (0.80) and NUtE (0.80) in the T1 condition. A significantly positive correlation of NUE was also recorded with PH in T1 and TPM, SL, and BMY in the T2 condition.

All the six physiological traits namely CC-1, CC-2, CC-3, NDVI-1, NDVI-2 and NDVI -3 showed highly significant and positive correlations with each other. Among NUE related traits, GPC had a significantly positive correlation with NHI, TNUp, and NUpE under both T1 and T2 conditions. Similarly, a highly significant and positive correlation of TNUp with NUpE and NUtE was observed in both conditions. NUpE has a highly significant and positive association with NUtE in T1 but negative in T2. For agro-morphological traits, NUE/grain yield showed highly positive correlations with DM, SPS, GPS, TGW and HI under both the nitrogen conditions. Traits SPS, GPS and TGW have a highly significant and positive correlation with each other under both conditions. DM and DH have highly significant and positive associations in T1 as well as T2 conditions. Besides these, highly significant and positive associations of SL with DM, TPM, SPS, GPS, TGW, HI; TPM with SPS, GPS, HI; HI with SPS, GPS, TGW and DH with PH were observed the in T1 condition whereas the similar correlation of DH and DM with SPS and HI were observed in the T2 condition. A highly significant but negative association of HI with BMY was also observed under both the nitrogen levels.

The NUE related traits showed highly significant and positive correlations with yield and its component traits among which GPC with TGW & HI; BMY with TNUp & NUpE and

NUtE with SPS, GPS, TGW & HI are prominent. All the physiological traits namely chlorophyll content and NDVI at booting, anthesis and grain filling stages showed highly significant and positive correlations with NUE related traits GPC, TNUp, and NUpE under both conditions. NUtE has similar associations with chlorophyll content at all the three stages under the T1 condition and with chlorophyll content and NDVI at the anthesis stage in the T2 condition. Considering yield and physiological traits, SPS, GPS, TGW, and HI showed highly significant and positive associations with all the six physiological parameters under both T1 and T2. Under both conditions, BMY showed significant negative associations with chlorophyll content at all three stages. Significantly positive associations of TPM with all the

Table.2: Phenotypic correlations among different morpho-physiological, yield, yield attributes, NUE and NUE related traits in 50 genotypes of bread wheat under T_1 (soil N + 50 kg Nha^{-1}) and T2 (Soil N + 100 kg Nha^{-1}) conditions

T1 T2	CC1	CC2	CC3	NDVI 1	NDVI 2	NDVI 3	DH	DM	PH	TPM	SL	AL	SPS	GPS	TGW	BY	HI	GPC	NHI	TNUp	NUpE	NUtE	NUE
CC 1	**1.00**	0.97**	0.98**	0.33**	0.43**	0.39**	0.14	0.07	-0.02	0.19	0.16	0.22*	0.21*	0.23*	0.54**	-0.23*	0.44**	0.38**	-0.06	0.25*	0.24*	0.21*	0.40**
CC 2	0.90**	**1.00**	0.97**	0.36**	0.46**	0.42**	0.16	0.10	0.02	0.15	0.17	0.21*	0.25*	0.27**	0.57**	-0.24*	0.47**	0.42**	-0.06	0.25*	0.24*	0.26**	0.45**
CC 3	0.82**	0.87**	**1.00**	0.35**	0.43**	0.40**	0.15	0.07	0.02	0.21*	0.15	0.24*	0.22*	0.24*	0.52**	-0.24*	0.44**	0.43*	-0.03	0.25*	0.24*	0.21*	0.41**
NDVI 1	0.38**	0.38**	0.45**	**1.00**	0.83**	0.88**	0.35**	0.34**	-0.08	-0.01	0.08	0.19	0.31**	0.31**	0.36**	0.08	0.24*	0.36**	-0.01	0.45**	0.44**	0.11	0.43**
NDVI 2	0.40**	0.39**	0.48**	0.56**	**1.00**	0.91**	0.37**	0.36**	-0.01	0.05	0.16	0.14	0.45**	0.44**	0.48**	-0.07	0.42**	0.34**	0.06	0.32**	0.31**	0.27**	0.51**
NDVI 3	0.43**	0.42**	0.52**	0.73**	0.64**	**1.00**	0.34**	0.34**	-0.06	0.06	0.15	0.20*	0.45**	0.43**	0.46**	-0.06	0.39**	0.47**	0.09	0.37**	0.37**	0.25*	0.51**
DH	0.12	0.11	0.06	-0.06	-0.07	-0.04	**1.00**	0.79**	0.16	-0.04	0.21*	-0.05	0.38**	0.38**	0.08	-0.27**	0.40**	0.13	0.07	-0.08	-0.08	0.42**	0.36**
DM	0.06	0.08	0.03	-0.07	-0.11	0.04	0.80**	**1.00**	-0.02	-0.12	0.18	0.00	0.26**	0.24*	0.05	-0.09	0.29**	0.14	0.28**	0.01	0.01	0.41**	0.40**
PH	0.11	0.06	0.04	0.05	0.01	0.00	0.27**	0.20*	**1.00**	-0.08	0.04	0.09	-0.01	-0.01	-0.21*	-0.04	-0.08	-0.08	-0.15	-0.10	-0.09	-0.09	-0.17
TPM	0.28**	0.27**	0.30**	0.31**	0.23*	0.40**	-0.06	0.02	0.09	**1.00**	0.01	0.02	0.18	0.17	0.13	-0.15	0.24*	0.19	0.03	0.09	0.07	0.14	0.23*
SL	0.46**	0.48**	0.39**	0.15	0.15	0.11	0.21*	0.29**	0.18	0.34**	**1.00**	0.19	0.17	0.19	0.06	0.06	0.07	-0.05	0.00	0.09	0.10	0.13	0.20*
AL	0.06	0.11	0.11	0.07	0.16	0.11	-0.18	-0.05	-0.13	0.08	0.09	**1.00**	-0.03	-0.01	0.06	0.23*	-0.10	0.14	-0.04	0.34**	0.34	-0.18	0.07
SPS	0.40**	0.41**	0.40**	0.32**	0.32**	0.31**	0.18	0.19	0.13	0.38**	0.55**	0.08	**1.00**	0.99**	0.33**	-0.26 **	0.47 **	0.14	0.01	0.01	0.04	0.43**	0.47**
GPS	0.42**	0.42**	0.39**	0.28**	0.37**	0.28**	0.18	0.18	0.15	0.40**	0.55**	0.13	0.96**	**1.00**	0.34**	-0.26 **	0.47 **	0.15	0.01	-0.00	0.00	0.44**	0.47**
TGW	0.52**	0.50**	0.60**	0.39**	0.34**	0.38**	-0.05	-0.03	0.12	0.17	0.38**	0.08	0.38**	0.35**	**1.00**	-0.33 **	0.59 **	0.42**	-0.03	0.21*	0.22*	0.36**	0.54 **
BY	-0.26**	-0.28**	-0.23*	-0.15	-0.11	-0.07	-0.16	0.08	-0.01	0.06	-0.09	0.06	-0.06	-0.06	-0.32**	**1.00**	-0.80 **	- 0.17	0.10	0.59 **	0.58 **	-0.62 **	-0.24*
HI	0.59**	0.62**	0.58**	0.39**	0.35**	0.39**	0.24*	0.18	0.19	0.43**	0.49**	-0.04	0.50**	0.48**	0.55**	-0.48 **	**1.00**	0.36**	0.02	-0.18	-0.17	0.83 **	0.74**
GPC	0.44**	0.44**	0.47**	0.31**	0.20*	0.33**	0.11	0.06	-0.02	0.15	0.21	0.14	0.16	0.15	0.36**	-0.36 **	0.32**	**1.00**	0.38**	0.23 *	0.24 *	0.23*	0.40**
NHI	-0.04	-0.09	0.01	-0.03	0.00	0.04	0.12	0.11	-0.03	0.06	-0.04	0.13	0.04	0.03	-0.16	0.28 **	-0.23*	0.29**	**1.00**	-0.17	-0.17	0.32*	0.19
TNUp	0.42**	0.46**	0.45**	0.32**	0.26**	0.39**	0.01	0.18	0.14	0.43**	0.38**	0.09	0.33**	0.32**	0.36**	0.33 **	0.53**	0.32**	-0.14	**1.00**	0.99 **	0.38 **	0.32 **
NUpE	0.43**	0.47**	0.46**	0.33**	0.24*	0.38**	0.03	0.18	0.14	0.43**	0.39**	0.07	0.32**	0.30**	0.36**	0.30 **	0.53**	0.33**	-0.13	0.99**	**1.00**	-0.37 **	0.32 **
NUtE	0.31**	0.31**	0.30**	0.19	0.22*	0.24*	0.23*	0.22*	0.17	0.41**	0.33**	-0.15	0.44**	0.42**	0.24**	-0.12	0.75**	-0.15	0.05	0.28**	0.29**	**1.00**	0.72**
NUE	0.48**	0.50**	0.49**	0.33**	0.30**	0.38**	0.18	0.26**	0.20*	0.52**	0.47**	-0.05	0.49**	0.47**	0.38**	0.10	0.82**	0.12	-0.06	0.79**	0.80**	0.80**	**1.00**

Highly significant & positive correlations
Highly significant & negative correlations
Significant & positive correlations
Significant & negative correlations
Positive correlations
Negative correlations

Correlation: T1- below diagonal, T2- Above diagonal

CC1, CC2, CC3 & NDVI1, NDVI2, NDVI3- Chlorophyll contents and NDVI at booting, anthesis and grain filling stages, respectively, DH- Days to heading, DM- Days to maturity, PH- Plant height, TPM- Number of productive tiller per meter, SL- Spike length, AL-Awn length, SPS-Number of spikelet's per spike, GPS-Number of grains per spike, TGW-Thousand grain weight, BY- Biomass yield, HI- Harvest index, GPC-Grain protein content, NHI- Nitrogen harvest index, TNUp-Total nitrogen uptake, NUpE-Nitrogen uptake efficiency, NUtE- Nitrogen utilization efficiency, NUE-Nitrogen use efficiency

physiological parameters and SL with chlorophyll content (CC-1, CC-2, CC-3) were observed in T1 only. In T2, significant and positive associations of DH and DM with NDVI and AL with chlorophyll content at different stages were observed.

3.4 Performance of genotypes for yield and NUE related traits

Based on GY data, the top 10 highest yielding genotypes and the least 10 poor grain yielding genotypes were identified under N50 conditions which were identified as N-insensitive (NIS) and N-sensitive (NS) genotypes, respectively as per Pujarula *et al*., (2021). Out of 10 NIS genotypes, four genotypes (UASBW13356, UASBW13358, UASBW13354 and UASBW13357) were common in the top 10 high yielding genotypes at the N100 level showing genotypic plasticity towards N50 and N100 conditions (Fig 2A). Similarly, out of 10 NS genotypes, 7 lines (KRL237, DTW2011-56, HI8730, DL153-2, UASBW13362, WH1021 and MP1293) were also included in the 10 least yielding genotypes in T2 (Fig. 2B). NUE and its associated traits also showed a wide range among genotypes under both T1 and T2 conditions (Table. 3). The top 10 promising genotypes for each trait were identified under both conditions. The common genotypes in both T1 and T2 conditions for NUE (UASBW13356, UASBW13358, UASBW13354, and UASBW13357), TNUp & NUpE (UASBW13356, PBW175, Raj1972, and WH1022), NHI (UASBW13362), NUtE (UASBW13356, UASBW13358, UASBW13354, and UASBW13364) and GPC (UASBW13356, UASBW13358, PBW175, K9107, HD2967, and GW322) were identified as potential donors. Further, the NutE by genotypes under T1 was compared with NUtE under T2 to determine their efficiency and their responsiveness to N by their classification into four groups (Fig.3) viz., N efficient responsive (NER), N efficient non-responsive (NENR), N inefficient responsive (NIR) and N inefficient non-responsive (NINR) as per Rengel and Graham (1995) and Worku *et al*., (2007). The average NUtE of all the 50 genotypes under T1 (30.16) and T2 condition (28.08) were considered a cut-off for the identification of genotypic efficiency and responsiveness.

Table. 3: Mean Performance for NUE traits under T_1 (soil N + 50 kg Nha^{-1}) and T2 (Soil N + 100 kg Nha^{-1}) conditions and stress indices in bread wheat genotypes

S No.	Genotypes	Gr Yield (q/ha)		NUE related traits								Stress indices		
				NUE		NUpE		NUtE		GPC		YSI	SSI	PR (%)
		T1	T2	T1	T2	T1	T2	T1	T2	T1	T2			
1	DBW 14	23.32	30.28	12.92	14.23	0.50	0.57	25.98	25.26	11.33	13.17	0.77	2.02	22.99
2	DL 153-2	20.60	28.64	11.39	13.45	0.42	0.48	27.12	27.99	11.58	11.61	0.72	2.46	28.08
3	DTW 2011-56	19.16	20.40	10.59	9.57	0.44	0.53	24.01	17.95	12.19	10.85	0.94	0.53	6.07
4	GW 2013-540	28.32	30.33	15.69	14.24	0.49	0.54	32.44	26.91	11.41	13.06	0.93	0.58	6.63
5	GW 322	28.78	31.98	15.96	15.02	0.50	0.48	31.84	31.19	13.04	13.26	0.90	0.88	10.00
6	HD 2189	24.60	29.03	13.63	13.61	0.47	0.49	29.39	27.92	10.56	11.56	0.85	1.34	15.24
7	HD 2733	29.77	35.54	16.44	16.66	0.54	0.63	30.84	26.60	12.84	12.78	0.84	1.42	16.24
8	HD 2967	31.25	37.90	17.29	17.78	0.54	0.59	32.59	30.64	13.07	13.45	0.82	1.54	17.54
9	HI 1500	28.94	30.23	16.01	14.17	0.53	0.48	30.73	32.22	12.32	12.56	0.96	0.37	4.26
10	HI8730	19.27	23.34	10.68	10.97	0.49	0.52	22.11	21.24	11.48	11.28	0.83	1.53	17.42
11	HPW251	26.88	30.61	14.88	14.37	0.51	0.48	29.11	30.28	12.74	13.33	0.88	1.07	12.17
12	K 9107	31.42	37.29	17.38	17.49	0.58	0.57	30.13	31.11	13.10	13.63	0.84	1.38	15.74
13	Kalyansona	30.83	33.26	17.05	15.62	0.57	0.55	30.21	28.66	12.98	12.97	0.93	0.64	7.31
14	KRL 237	16.87	25.51	9.36	11.96	0.40	0.51	23.41	23.32	12.16	12.25	0.66	2.97	33.86
15	KRL1-4	32.15	34.28	17.81	16.08	0.58	0.54	31.06	29.54	11.85	12.22	0.94	0.55	6.21
16	MP 1293	25.24	27.97	13.99	13.12	0.50	0.56	28.29	23.49	11.21	12.02	0.90	0.86	9.75
17	MP 4010	26.42	26.72	14.60	12.54	0.53	0.58	27.61	21.78	12.42	12.17	0.99	0.10	1.12
18	NP846	27.90	27.80	15.46	13.07	0.59	0.56	26.44	23.32	12.72	13.22	1.00	-0.03	-0.37
19	PBW 175	32.36	30.83	17.89	14.49	0.62	0.62	28.89	23.31	12.92	13.63	1.05	-0.44	-4.97

20	PBW 343	28.80	38.45	15.93	18.04	0.51	0.52	31.77	34.92	13.06	13.20	0.75	2.20	25.08
21	RAJ 1972	29.96	32.89	16.57	15.41	0.63	0.62	26.55	24.96	12.55	13.70	0.91	0.78	8.90
22	RAJ 4248	27.93	32.93	15.48	15.42	0.56	0.59	28.01	26.21	12.32	12.76	0.85	1.33	15.16
23	UAS 304	33.87	34.43	18.73	16.15	0.57	0.53	32.98	30.64	11.62	12.20	0.98	0.14	1.63
24	UAS 323	29.25	35.55	16.22	16.68	0.53	0.58	30.46	29.04	10.99	12.68	0.82	1.55	17.71
25	WH 147	25.29	30.17	14.02	14.14	0.45	0.42	31.08	34.01	11.95	11.76	0.84	1.42	16.16
26	WH 542	29.58	32.32	16.37	15.17	0.47	0.55	34.99	27.84	10.70	11.67	0.92	0.75	8.49
27	WH 1021	22.83	26.87	12.61	12.58	0.47	0.45	26.99	27.93	12.21	12.87	0.85	1.32	15.04
28	WH 1022	30.47	36.47	16.85	17.10	0.58	0.62	29.42	27.84	12.94	13.17	0.84	1.44	16.46
29	UASBW13354	34.20	38.76	18.90	18.19	0.57	0.53	33.10	34.61	12.52	12.95	0.88	1.04	11.75
30	UASBW13355	29.10	39.06	16.09	18.32	0.51	0.56	31.47	32.90	12.70	13.60	0.75	2.24	25.50
31	UASBW13356	38.91	40.36	21.54	18.95	0.62	0.59	35.15	32.27	13.29	13.69	0.96	0.32	3.60
32	UASBW13357	33.34	38.08	18.47	17.86	0.61	0.58	30.61	30.86	12.75	12.59	0.88	1.09	12.44
33	UASBW13358	38.12	40.12	21.11	18.83	0.62	0.54	34.02	35.17	13.04	13.34	0.95	0.44	4.98
34	UASBW13359	33.60	35.43	18.59	16.63	0.58	0.55	32.15	33.40	12.99	12.71	0.95	0.46	5.19
35	UASBW13360	29.39	31.48	16.25	14.78	0.56	0.54	29.29	27.84	12.73	13.08	0.93	0.58	6.65
36	UASBW13361	29.09	33.50	16.11	15.71	0.51	0.59	31.83	26.67	10.67	12.86	0.87	1.16	13.16
37	UASBW13362	22.19	26.08	12.28	12.22	0.46	0.54	26.98	22.67	12.17	12.80	0.85	1.31	14.90
38	UASBW13363	27.70	28.98	15.32	13.61	0.49	0.47	31.68	28.76	11.85	12.25	0.96	0.39	4.42
39	UASBW13364	32.02	34.53	17.71	16.17	0.52	0.52	34.38	31.28	12.04	10.90	0.93	0.64	7.26
40	UASBW13365	25.81	28.97	14.31	13.58	0.50	0.53	29.06	25.66	11.80	12.54	0.89	0.96	10.90
41	UASBW13366	29.44	33.22	16.33	15.61	0.47	0.58	33.40	27.22	11.14	12.41	0.89	0.99	11.38
42	UASBW13367	31.05	32.40	17.20	15.23	0.53	0.55	32.68	28.21	11.18	12.97	0.96	0.36	4.14
43	UASBW12758	26.42	31.17	14.64	14.67	0.49	0.59	30.22	24.77	12.15	12.22	0.85	1.34	15.25
44	UASBW10227	28.57	29.13	15.80	13.65	0.54	0.54	29.32	25.18	12.26	11.15	0.98	0.17	1.91
45	UASBW10453	30.44	35.44	16.85	16.64	0.54	0.58	31.57	29.35	11.04	12.04	0.86	1.24	14.11
46	UASBW11948	29.73	34.42	16.42	16.13	0.51	0.63	31.94	25.67	11.27	11.77	0.86	1.19	13.61
47	UASBW12380	31.00	34.25	17.13	16.06	0.56	0.60	30.89	27.04	12.03	12.13	0.91	0.83	9.49
48	UASBW12876	29.80	33.01	16.48	15.47	0.52	0.48	31.72	32.54	11.14	12.42	0.90	0.85	9.72
49	UASBW12877	28.58	32.57	15.80	15.28	0.55	0.54	29.03	28.75	11.36	12.58	0.88	1.08	12.26
50	UASBW12878	31.84	33.16	17.62	15.54	0.53	0.58	33.36	27.13	10.95	12.13	0.96	0.35	3.96
	Mean	28.65	32.32	15.85	15.16	0.52	0.54	30.16	28.08	12.06	12.56	0.89	0.99	11.33
	CD @ 5%	5.18	3.94	2.85	1.70	0.06	0.09	4.03	4.40	1.34	1.26			

NUE-Nitrogen use efficiency, NUpE-Nitrogen uptake efficiency, NUtE-Nitrogen utilization efficiency, GPC-Grain protein content, YSI-Yield stability index, SSI, Stress susceptibility index, PR- Percent reduction under stress

for N use. Above-average genotypes under T1 were considered efficient (E) whereas below-average genotypes were considered inefficient (I). Similarly, in T2, above-average genotypes were considered responders(R), and below-average genotypes are considered non-responders (NR). In this classification, N was prefixed to indicate nitrogen. Considering together the category in T1 and T2, it was observed that efficient genotypes are higher in the utilization of absorbed N over inefficient genotypes. In this way 20 genotypes *viz*., GW322, HD2967, HI1500, Kalyansona, KRL1-4, PBW343, UAS304, UAS323, UASBW13354, UASBW13355, UASBW13356, UASBW13357, UASBW13358, UASBW13359, UASBW13363, UASBW13364, UASBW13367, UASBW10453, UASBW12876, WH147 were categorised as efficient and responsive whereas 18 genotypes namely, HD2189, DBW14, DL153-2, DTW2011-56, HI8730, KRL237, MP1293, MP4010, NP846, PBW175, RAJ1972, RAJ4248, UASBW13360, UASBW13362, UASBW13365, UASBW10227, WH1021, and WH1022 were found as inefficient and non-responsive. Similarly, genotypes GW2013-540, HD2733, UASBW13361, UASBW12758, UASBW11948, UASBW12380, and UASBW12878, WH542, UASBW13366 were classified as efficient but non-responsive and HPW251, K9107, and UASBW12877 were categorised as in-efficient but responsive genotypes to N fertilisers.

3.5 Stress tolerance indices for NUE

The pooled data of grain yield in T1 and T2 for all the 50 genotypes were used to estimate different stress related indices for the identification of promising genotypes that have the least effects of stress conditions, i.e., reduced nitrogen levels (Table.3). Three different indices namely yield stability index (YSI), stress susceptibility Index (SSI), and percent reduction under stress (PR) were estimated among which higher values of YSI and lower values of SSI and PR are desirable for the identification of promising genotypes under stress conditions. Genotypes showed a mean YSI of 0.89 with a range of 0.66 to 1.05 whereas the mean SSI was 0.99 with a range of -0.44 to 2.97. PR among genotypes was 11.33 ranging from -4.97 to 33.86 among all the genotypes, exotic lines showed better performance for YSI (0.90), SSI (0.86), and PR (9.84) in comparison to indigenous Indian germplasm lines which showed mean YSI, SSI, and PR of 0.88, 1.10 and 12.5 respectively. The results indicated that two indigenous genotypes namely PBW175 and NP846 showed higher yield at reduced nitrogen levels which is also supported by negative SSI and PR values in PBW175 (-0.44 & -4.97) and NP846 (-0.03 & -0.37), respectively. The perusal of results of YSI, SSI, and PR indicated that PBW175, NP846, MP4010, UAS304, and UASBW10227 were promising genotypes that were least affected for grain yield under reduced nitrogen doses. In addition, the indigenous germplasm lines, lines, HI1500, DTW2011-56, KRL1-4, GW2013-540, and exotic germplasm lines UASBW13356, UASBW12878, UASBW13367, UASBW13363, UASBW13358, and UASBW13359 showed promising performance for these three stress indices.

3. Discussion

Nitrogen (N) is often a limiting factor for crop yield and is therefore considered an essential macronutrient required for plant growth. Globally, the adoption of N-responsive cultivars and extensive application of N fertilizer has augmented food production in the past 50 years of agriculture. This excessive application of N fertilizer is increasing the cost of cultivation and thereby reducing the net profitability at the farm level in addition to negative impacts on the environment (Raun *et al*., 2002; Rothstein, 2007; Pathak *et al*., 2011; Ali *et al*., 2018; Hawkesford and Griffiths, 2019). The present investigation was driven by a worldwide willingness, especially from a huge number of small and marginal farmers in developing countries, to understand genotype behaviour for the identification and development of genotypes (without compromising GY) with high NUE under low N conditions. As demand for wheat grain is still rising, there is a need to boost productivity and production for the amount of N applied and therefore, identification of genotypes with better NUE is crucial. The immediate aim should, therefore, be to exploit the available variability for NUE in wheat cultivars through the appropriate breeding procedure.

The present study aimed to understand the variability and correlations for NUE traits with various yield and physiological parameters and identify the promising genotypes based on various parameters of nitrogen sensitivity, response to N fertilizers, and susceptibility indices to low N availability. Précised phenotyping under low N input is challenging and influenced by the genotype (G), environment (E), and G × E interactions (Chen *et al*., 2014; Rao *et al*., 2018) which makes it difficult to identify nitrogen insensitive genotypes under low N conditions at field level. Presently, very few wheat breeding programs are targeting the development of low N tolerance traits which are a must for sustainable agriculture with minimal negative impacts on the environment.

In this study, a total of 50 diverse wheat genotypes collected from indigenous germplasm and international nurseries from CIMMYT were characterized for 24 yield, yield component, physiological, and NUE related traits at two different N levels under field

conditions. Analysis of variance indicated highly significant variation among genotypes at both treatments for all the traits studied and this existence of significant differences for the different attributes is very helpful to improve the trait of interest. Diverse responses have been observed among the genotypes for all the traits across N levels, despite similar growth conditions and an equal amount of N fertilizer in a given N level. The genotypic variations observed purely reveal trait specific genotype plasticity. These results concur with other reported field experiments on wheat (Sial *et al*., 2005; Belete *et al*., 2018).

Wheat is more responsive to a higher dose of nitrogen whereas, sensitive to less nitrogen availability like most cereal crops. Less soil N availability leads to reduced plant vegetative growth especially tillering which results in reduced grain yield. (Kaur *et al*., 2015). In this study, reduction in tillering and ultimately yield reduction to the tune of approx. 11.3% was observed at reduced N levels as compared to the recommended dose. Similarly, the low N availability is expressed phenotypically in the form of low chlorophyll content as indicated by lower SPAD and NDVI values which might be due to less N availability in the leaves at lower N levels. Similar results of increased chlorophyll content by increasing N application were reported by Kitajima and Hogan (2003), and Pramanik and Bera (2013). Almost all the traits studied except NDVI-1, HI, NUE, NUtE and NHI, showed reduced performance at lower N doses which reflects the genotypic sensitivity to low N availability. The traits *viz*., CC-1, CC-2, SPS, TGW, GY, BMY, and TNUp, were identified as the most sensitive traits to reduced N supply (Table 3). Paponov and Engels (2005) also elaborated the effect of low N supply on the grain filling process. When N supply was low, the remobilization of N from the tillers and leaves was efficient and converted into grain N indicating better NUtE. This suggested that few genotypes have the potential to use nitrogen more efficiently and thus exhibit high NUE. The NHI indicates the level of efficiency of plants to use acquired total N for grain formation (He *et al*., 2017) for which significant genotypic variation was observed at both the N levels. Varied response of genotypes to NUpE, NUtE, and NHI was observed by Ortiz-Monasterio *et al*., (1997); Le Gouis *et al*., (2000); Brancourt-Hulmel *et al*., (2003); Muurinen *et al*., (2007); Chen *et al*., (2011) and Monostori *et al*., (2017) which are in tune with the performance of NUE related traits at varying N levels in the present study.

To assess the nature and magnitude of diversity among the genotypes, the phenotypic and genotypic coefficients of variation were estimated. In general, higher PCV values were observed as compared to GCV values at both the nitrogen levels. High PCV and GCV were observed for harvest index whereas other major yield traits and NUE related traits showed moderate values. Similarly, previous studies have established significant genetic variation for NUE-related traits in wheat (Yousef *et al*., 2008; Kalimullah *et al*., 2012).

In general, moderate to high heritability and was recorded under both nitrogen levels. The heritability values in a broad sense ranged from 34.05% to 86.84% in T1 and 37.81% to 93.84% in T2. High heritability (>60%) were observed for CC-3, DH, DM, SPS, GY, BMY, HI, NUE, and NUtE under both the conditions. A similar trend was also observed for yield and NUE related traits by Majumder *et al*. (2008) and Nikolic *et al*. (2013). High heritability is useful in laying importance in choice for such traits during selection.

In the present study, nitrogen use efficiency was measured as grain yield divided by available N (soil N + fertilizer N) due to which the correlation of NUE and grain yield at both levels of nitrogen treatments showed similar results. The positive and highly significant correlated response of NUE with DM, HI, TGW, GPS, SPS, CC-1, CC-2, CC-3, NDVI-1, NDVI-2, NDVI -3 TNUp, NUpE, and NUtE under both the condition suggested improvement of these traits for better NUE in wheat genotypes (Rangare *et al*., 2010; Baranwal *et al*., 2012).

Interestingly highly significant and positive associations between physiological traits and NUE related traits were also observed. Similar associations of NUE related traits by Gorjanovic *et al*., (2011); Ayadi *et al*., (2012); Beche *et al*., (2014); Kaur *et al*. (2015), and Monostori *et al*. (2017) indicated that these characters should be considered as NUE components in crop breeding that can be harnessed for higher grain yields under both low and high N fertility conditions.

Across the year on average, GY ranged from 16.87 to 38.91 q/ha (N50) and 20.4 to 40.36q/ha (N100), indicating the genotypic variability in GY at different N regimes. In general, increased GY was correlated with the enhanced N application rate, which might be due to sufficient nitrogen availability. Reduced GY by 10% under low N conditions compared to normal conditions was also reported by Šarčević *et al*., (2014) and Hawkesford and Griffiths (2019) in wheat. Remarkably top 10 highest yielding genotypes were identified under N50 conditions as N-insensitive (NIS) among which four genotypes (UASBW13356, UASBW13358, UASBW13354, and UASBW13357) were common in the top 10 high yielding genotypes at the N100 level showing their genotypic plasticity towards varying N levels. These genotypes may be potential genetic resources to breed for tolerance to low N conditions. On the other hand, the top 10 high yielding genotypes in N100 can have the best acceptances for cultivation where soils are fertile and following the ideal N levels for cultivation and could be further used to improve GY, along with higher NUE. Several studies suggested the utilization of N efficient lines with enhanced GY in the farmer fields which may help to reduce fertilizer input as well as increase the profitability of farm operations (Würschum, 2012; Vijayalakshmi *et al*., 2015; He *et al*., 2017). Similarly, selected NIS lines with high NUE will certainly play a role in reducing environmental pollution and could increase economic profit for farmers.

The present study indicated that NUE and its associated traits also showed a wide range among genotypes for all the six NUE related traits. The higher frequency of genotypes in medium to high class based on NUE parameters suggests the potentiality of these tested genotypes (Fig.4). The best performing 10 entries were identified for each trait under T1 and T2 conditions among which common genotypes under both the conditions were identified as promising trait specific genetic resources. Further, UASBW13356, UASBW13358, UASBW13354, PBW 175, Raj 1972, and WH 1022 were found promising for multiple NUE traits and may be utilized extensively for NUE improvement programme in wheat. Determination of the genetic variations for NUE related traits is essential for the selection of efficient genotypes that can be used further in breeding programs to develop low N tolerant material. The concept of genotype grouping is used widely in nutrient use efficiency (Fageria and Baligar, 2003). Among NUE related traits, nitrogen utilization efficiency has the most significance which is defined as the genotype ability to assimilate and remobilize N ultimately to produce the GY (Rengel and Graham, 1995; Fageria *et al*., 2008). Large genotypic variations in NUtE have been reported under field/pot screening in various wheat genotypes and other crops (Bouchet *et al*., 2014; Ma *et al*., 2015). Based on NUtE efficiency data in T1 (N50) versus T2 (N100), above and below average genotypes under T1 were considered efficient and inefficient, respectively whereas, above and below average genotypes in T2 were considered as responders, and non-responders, respectively.

Based on NUtE in T1(N50), 29 efficient (E) and 21 inefficient (I) genotypes were identified whereas 23 responsive (R) and 27 non-responsive (NR) genotypes were identified based on NUtE in T2 (N100), Considering together, all the 50 genotypes were classified into four groups viz., NER (20), NENR (9), NIR (03) and NINR (18) as per Rengel and Graham (1995) and Worku *et al*., (2007). The NENR genotypes showed a progressive performance under low N which may enable breeders to develop efficient genotypes under low input environments. The remaining 20 NER genotypes were most desirable that exhibited a progressive response to increased N availability. These NER genotypes could be the prospective targets for selection for the genetic improvement of wheat for better N utilization. Interestingly, among the 10 NIS genotypes, 8 genotypes namely, UASBW13356, UASBW13358, UASBW13354, UAS304, UASBW13359, UASBW13357, KRL1-4, and UASBW13364 were N efficient and responsive which showed progressive performance in terms of efficient and responsive use of nitrogen. High GY per unit of N consumption could be obtained from the genotypes having more NUtE (Moll *et al*., 1982), and thus, breeding for efficient genotypes under low N could be progressed with high NUtE (Fageria, 2014; He *et al*., 2017). Similarly, inefficient but responsive genotypes (HPW251, K9107, and UASBW12877) can be used in breeding programs for further improvement. The rest of the inefficient, nonresponsive genotypes are less desirable from the NUE point of view. Overall, it was found that the efficient genotypes are higher in the utilization of absorbed nutrients than inefficient genotypes.

The differential behaviour of genotypes under stress was studied by estimation of three different stress related indices for the identification of promising genotypes that have the least effects of stress conditions, *i.e*., reduced nitrogen levels. Among these indices, higher values of YSI and lower values of SSI and PR were desirable for the identification of promising genotypes under stress conditions. A wide range was observed among genotypes for these indices and exotic genotypes showed more desirable indices. Based on these indices, the indigenous germplasm lines, HI1500, DTW2011-56, KRL1-4, GW2013-540, and exotic germplasm lines UASBW13356, UASBW12878, UASBW13367, UASBW13363, UASBW13358, and UASBW13359 were found promising. These indices also indicated PBW175, NP846, MP4010, UAS304, and UASBW10227 as the least affected genotypes for grain yield under reduced nitrogen doses.

4. Conclusions

Improving NUE is pivotal for sustainable crop growth and yield, especially under low nitrogen soils on which depend majority of poor farmers in most developing countries, especially that in South Asia. Likewise, improving crop productivity using N fertilization is important for achieving climate resilience. Nevertheless, the genetic improvement of NUE depends on the nature and extent of variation among the germplasm. This study aimed to derive morphologic and agronomical traits associated with NUE in a set of 50 diverse lines under different N levels. Although the nitrogen limitation resulted in the reduction of GY, extensive genetic variations for grain yield, NUE, and their associated traits were noted among the genotypes at both levels of nitrogen. The associations between yield and NUE traits indicated avenues for wheat improvement concerning enhanced NUE and thereby yield. Different selection parameters for the selection of nitrogen-insensitive genotypes, nitrogen efficient and responsive genotypes, and stress tolerance due to low N availability have identified UASBW13356, UASBW13358, UASBW13354, UASBW13357, and KRL1-4 as promising genotypes that showed their inherent genotypic plasticity toward N application. The findings suggest that genotypes with more yield and high to moderate NUtE can be used as parents for the breeding of N efficient genotypes for marginal agro-ecologies.

Furthermore, these nitrogen-use efficient genotypes can be further tested on large scale to know their stability for their release as a variety so that farmers can get the advantage of low input cost. These genotypes have the potential to decrease environmental pollution and high economic costs associated with excess N fertilizer. Additionally, these are the best suggested genotypes for organic farming due to their inherent potentiality of low input response which may also help the vast number of small and marginal farmers for optimizing the use of fertilizer inputs for economic and environmentally sustainable food production.

Author Contributions

The first and second authors contributed equally to the manuscript. Suma S Biradar- Data curation, Methodology, Formal analysis, Visualization, Writing-original draft, **Mahalaxmi K Patil**- Conceptualization, Data curation, Methodology, Investigation, Formal analysis & Writing-original draft; **SA Desai**- Conceptualization, Methodology, Supervision, Writing - review & editing; **SK Singh**- Formal analysis, Writing - review & editing; **V Rudra Naik**- Conceptualization, Visualization, Writing - review & editing; **Kumar Lamani**- Methodology; Investigation, **Arun K Joshi**- Resources, Visualization. All authors have read and approved the final manuscript.

Declaration of interests

The authors declare that they have no known competing financial interests or personal relationships that could have appeared to influence the work reported in this paper.

Acknowledgments

We are thankful to the technical staff at Dr Sanjaya Rajaram Wheat Research Laboratory for data collection at the experimental site and laboratory technicians for Nitrogen (N) analysis in different samples.

References

Ali, J., Jewel, Z.A., Mahender, A., Anandan, A., Hernandez, J., Li, Z., 2018. Molecular genetics and breeding for nutrient use efficiency in rice. Int. J. Mol. Sci. 19, 1-27.

Anbessa, Y., Juskiw, P., Good, A., Nyachiro, J., and Helm, J., 2009. Genetic variability in nitrogen use efficiency of spring barley. Crop Sci. 49, 1259–1269. doi: 10.2135/cropsci 2008.09.0566

Ayadi, S., Karmous, C., Hammami, Z., Tamani, N., Yoissef, T., Esposito, S., Rezgui S., 2012. Genetic variability of nitrogen use efficiency components in Tunisian improved genotypes and landraces of durum wheat. Agril. Sci. Res. J. 2(11), 591-601.

Baranwal, D.K., Mishra, V.K., Vishwakarma, M.K., Yadav, Punam S., Arun, B., 2012. Studies on genetic variability, correlation and path analysis for yield and yield contributing traits in wheat (*T. aestivum L*. em Thell.). Plant Archives 12 (1):99-104

Barraclough, P.B., Howarth, J.R., Jones J., Lopez-Bellido, R., Parmar, S., Shepherd, C.E., Hawkesford, M.J., 2010. Nitrogen efficiency of wheat: Genotypic and environmental variation and prospects for improvement. Eur. J. Agron. 33, 1-11. doi: 10.1016/j.eja.2010.01.005

Beche, E., Benin, G., Lemes, C., Munaro, L., Marchese, J., 2014. Genetic gain in yield and changes associated with physiological traits in Brazilian wheat during the 20th century. Eur. J. Agron. 61, 49–59. 10.1016/j.eja.2014.08.005.

Belete, F., Dechassa, N., Molla, A., and Tana, T., 2018. Effect of nitrogen fertilizer rates on grain yield and nitrogen uptake and use efficiency of bread wheat (*Triticum aestivum* L.)

varieties on the Vertisols of central highlands of Ethiopia. Agric. Food Secur., 7, 1–12. doi:10.1186/s40066-018-0231-z

Borrell, A., Garside, A., Fukai, S., Reid, D., 1998. Season, nitrogen rate, and plant type affect nitrogen uptake and nitrogen use efficiency in rice. Aust. J. Agric. Res. 49, 829–843. doi: 10.1071/A97057

Bouchet, A.S., Nesi, N., Bissuel, C., Bregeon, M., Lariepe, A., Navier, H., Ribière, N., Orsel, M., Grezes-Besset, B., Renard, M., Laperche, A., 2014. Genetic control of yield and yield components in winter oilseed rape (Brassica napus L.) grown under nitrogen limitation. Euphytica 199, 183–205. doi: 10.1007/s10681-014-1130-4

Bouslama, M., Schapaugh, W.T., 1984. Stress Tolerance in Soybean. Part 1: Evaluation of Three Screening Techniques for Heat and Drought Tolerance. Crop Sci. 24, 933-937. https://doi.org/10.2135/cropsci1984.0011183X002400050026x

Brancourt-Hulmel, M., Goussinault, D., Lecomte, C., Berard, P., LeBuanec, B., Trottet, M., 2003. Genetic improvement of agronomic traits of winter wheat cultivars release in France from 1946 to 1992. Crop Sci. 43, 37-45.

Burton, G.W., 1952. Quantitative inheritance in grasses.Proc. Int. Grassland Congr. 1, 277-283.

Chen, B., Xu, K., Li, J., Li, F., Qiao, J., Li, H., Gao, G., Yan, G., Wu, X., 2014. Evaluation of yield and agronomic traits and their genetic variation in 488 global collections of *Brassica napus* L. Genet. Resour. Crop Evol. 61, 979–999. doi: 10.1007/s10722-014- 0091-8

Chen, C., Han, G., He, H., Westcott, M., 2011. Yield, protein, and remobilization of water soluble carbohydrate and nitrogen of three spring wheat cultivars as influenced by nitrogen input. Agron. J. 103, 786-795.

Choukan, R., Taherkhani, T., Ghanadha, M.R., Khodarahmi, M., 2006. Evaluation of drought tolerance in grain maize inbred lines using drought tolerance indices. Iranian Journal of Crop Sci. 8(1), 79-89.

Ciampitti, I.A., Vyn, T.J., 2012. Physiological perspectives of changes over time in maize yield dependency on nitrogen uptake and associated nitrogen efficiencies: A review. Field Crops Res. 133, 48–67. doi: 10.1016/j.fcr.2012. 03.008

Cormier, F., Foulkes, J., Hirel, B., Gouache, D., -Loccoz, Y. M. and Le Gouis, J., 2016, Breeding for increased nitrogen-use efficiency: a review for wheat (*T. aestivum* L.). Plant Breed. 135, 255–278. https://doi.org/10.1111/pbr.12371

Cormier, F., Le Gouis, J., Dubreuil, P., Lafarge, S. and Praud, S., 2014, A genome-wide identification of chromosomal regions determining nitrogen use efficiency components in wheat (*Triticum aestivum* L.). Theor. Appl. Genet. 127(12), 2679-2693. doi: 10.1007/s00122-014-2407-7

Fageria, N.K., 2014. Nitrogen harvest index and its association with crop yields. J. Plant Nutr., 37, 795–810. doi: 10.1080/01904167.2014.881855

Fageria, N.K., Baligar, V.C., 2003. Methodology for evaluation of lowland rice genotypes for nitrogen use efficiency. J. Plant Nutr. 26, 1315–1333. doi: 10.1081/PLN-120020373

Fageria, N.K., Baligar, V.C., Li, Y.C., 2008. The role of nutrient efficient plants in improving crop yields in the twenty first century. J. Plant Nutr. 31, 1121–1157.doi:10.1080/01904160 8021160 68

FAO. 2008. FAO fertilizer and plant nutrition bulletin: Guide to laboratory establishment for plant nutrient analysis. FAO, Rome, Italy, p. 203.

FAO. 2019. World fertilizer trends and outlook to 2022. Pp. 40

Fischer, R.A., Maurer, R., 1978. Drought Resistance in Spring Wheat Cultivars. I Grain Yield Responses. Aust. J. Agric. Res. 29, 897-912

Gaju, O., Allard, V., Martre, P., Snape, J.W., Heumez, E., Le Gouis, J., Bogard, M., Griffiths, S., Orford, S., Hubbart, S., Foulkes, M.J., 2011. Identification of traits to improve the nitrogen-use efficiency of wheat genotypes. Field Crops Res. 123, 139–152. doi: 10.1016/j.fcr.2011.05.010

Good, A.G., Shrawat, A.K., Muench, D.G., 2004). Can less yield more? Is reducing nutrient input into the environment compatible with maintaining crop production? Trends Plant Sci. 9, 597–605. doi: 10.1016/j.tplants.2004. 10.008

Gorjanovic, B.M., Brdar, J., Balalic, M.K., 2011. Phenotypic variability of bread wheat genotypes for nitrogen harvest index. Genetika 43(2), 419-426.

Hakeem, K.R., Chandna, R., Ahmad, A., Qureshi, M.I., Iqbal, M., 2012. Proteomic analysis for low and high nitrogen-responsive proteins in the leaves of rice genotypes grown at three nitrogen levels. Appl. Biochem. 168, 834–850. doi: 10.1007/s12010-012-9823-4

Hanson, C.H., Robinson, H.F., Comstock, R.E., 1956. Biometrical studies in yield of segregating population of Korean lespedeza. Agronomy J. 48, 214–318.

Hawkesford, M.J., 2014. Reducing the reliance on nitrogen fertilizer for wheat production. J. Cereal Sci. 59, 276–283. doi: 10.1016/j.jcs.2013.12.001

Hawkesford, M.J., 2017. Genetic variation in traits for nitrogen use efficiency in wheat. J. Exp. Bot. 68, 2627–2632. doi: 10.1093/jxb/erx079

Hawkesford, M.J., Griffiths, S., 2019. Exploiting genetic variation in nitrogen use efficiency for cereal crop improvement. Curr. Opin. 49, 35–42. doi: 10.1016/ j.pbi.2019.05.003

Hawkesford, M.J., Riche, A.B., 2020. Impacts of GxExM on Nitrogen Use Efficiency in Wheat and Future Prospects. Front. Plant Sci., 11, 1157.

He, H., Yang, R., Li, Y., Ma, A., Cao, L., Wu, X., Chen, B., Tian, H., Gao, Y., 2017. Genotypic variation in nitrogen utilization efficiency of oilseed rape (*Brassica napus*) under contrasting N supply in pot and field experiments. Front. Plant Sci. 8, 1825. doi: 10.3389/fpls. 2017.01825

Hickman, J.E., Palm, C.A., Mutuo, P., Melillo, J.M., Tang, J., 2014. Nitrous oxide (N_2O) emissions in response to increasing fertilizer addition in maize (*Zea mays* L.) agriculture in western Kenya. Nutr. Cycling Agroecosyst, 100, 177–187. doi: 10.1007/s10705-014-9636-7

Hirel, B., Le Gouis, J., Ney, B., Gallais, A., 2007. The challenge of improving nitrogen use efficiency in crop plants: towards a more central role for genetic variability and quantitative genetics within integrated approaches. J. Exp. Bot. 58, 2369–2387. doi: 10.1093/jxb/erm097

Inthapanya, P., Sihavong, P., Sihathep, V., Chanphengsay, M., Fukai, S., Basnayake, J., 2000. Genotype differences in nutrient uptake and utilisation for grain yield production of rainfed lowland rice under fertilized and non-fertilized conditions. Field Crops Res. 65, 57–68. doi: 10.1016/S0378-4290(99)00070-2

Kalimullah, Khan, S.J., Irfaq M., Rahman, H.U., 2012. Genetic variability, correlation and diversity studies in bread wheat (*Triticum aestivum* L.) germplasm. J. Anim. Plant Sci. 22(2), 330-333.

Kaur, G., Asthir, B., Bains, N.S., 2015. Nitrogen levels effect on wheat nitrogen use efficiency and yield under field condition. Afr. J. Agric. Res. 10(23), 2372-2377.

Kiran, T.V., Vijayalakshmi, P., Rao, Y.V., Swamy, K.N., Kondamudi, R., Srikanth, B., Rao, I.S., Babu, M.S., Jaldhani, V., Prema Latha, D., Neeraja, C.N., Surekha, K., Rao, P.R., Subrahmanyam, D., Subbarao, L.V., Voleti, S.R., 2016. Effects of nitrogen limitation on antioxidant enzymes, chlorophyll content and grain yield of rice genotypes. Asian Res. J. Agri. 1, 1–10.doi: 10.9734/arja/ 2016/28503

Kitajima, K., Hogan, K.P., 2003. Increases of chlorophyll a/b ratios during acclimation of tropical woody seedlings to nitrogen limitation and high light. Plant Cell Environ. 26, 857–865. doi: 10.1046/j.1365-3040.2003.01017.x

Ladha, J.K., Kirk, G.J.D., Bennett, J., Peng, S., Reddy, C.K., Reddy, P.M., Singh, U., 1998. Opportunities for increased nitrogen-use efficiency from improved lowland rice germplasm. Field Crops Res. 56, 41–71. doi: 10.1016/S0378-4290(97)00123-8

Ladha, J.K., Tirol-Padre, A., Reddy, C.K., Cassman, K.G., Verma, S., Powlson, D.S., van Kessel, C., Richter, D.D.B., Chakraborty, D., Pathak, H., 2016. Global nitrogen budgets in cereals: A 50-year assessment for maize, rice, and wheat production systems. Sci. Rep. 6,19355. doi: 10.1038/ srep19355

Lassaletta, L., Billen, G., Grizzetti, B., Anglade, J., Garnier, J., 2014. 50 year trends in nitrogen use efficiency of world cropping systems: the relationship between yield and nitrogen input to cropland. Environ. Res. Lett. 9,105011. doi: 10.1088/1748-9326/9/10/105011

Le Gouis, J., Beghim, D., Heumez, E. and Pluchard, P., 2000. Genetic differences for nitrogen up take and nitrogen utilization efficiencies in winter wheat. Eur. J. Agron. 12, 163-173. doi: 10.1016/S1161-0301(00)00045-9

Le Gouis, J., Gaju, O., Hubbart, S., Allard, V., Orford, S., Heumez, E., Bogard, M., Griffiths, S., Wingen, L., Semenov, M., Martre, P., Snape, J., Foulkes, J., 2010. Genetic improvement for an increased nitrogen use efficiency in wheat. Asp. Appl. Biol. 105, 151-158.

Lush, J.L., 1940. Intra-class correlations or regression of offspring on dam as a method of estimating heritability of characteristics. Am. Soc. Anim. Prod. 33, 293–301

Ma, B.L., Biswas, D.K., Herath, A.W., Whalen, J.K., Ruan, S.Q., Caldwell, C., Earl, H., Vanasse, A., Scott, P., Smith, D.L., 2015. Growth, yield, and yield components of canola as affected by nitrogen, sulfur, and boron application. J. Plant Nutr. Soil Sci. 178, 658–670. doi: 10.1002/jpln.201400280

Majumder B., Mandal, B., Bandyopadhyay, P.K., Gangopadhyay, A., Mani, P.K., Kundu, A.L., Mazumdar, D., 2008. Organic amendments influence soil organic carbon pools and rice–wheat productivity. Soil Sci. Soc. Am. J. 72(3), 775-785. doi:10.2136/sssaj 2006.0378

Maman, N., Mason, S.C., Lyon, D.J., 2006. Nitrogen rate influence on pearl millet yield, nitrogen uptake, and nitrogen use efficiency in Nebraska. Commun. Soil Sci. Plant Anal.37, 127–141. doi: 10.1080/00103620500406112

Moll, R.H., Kamprath, E.J., and Jackson, W.A., 1982. Analysis and interpretation of factors which contribute to efficiency of nitrogen utilization. Agron J. 74, 562-564. doi: 10.2134/ agronj 1982.00021962007400030037x

Monostori, I., Árendás, T., Hoffman, B., Galiba, G., Gierczik, K., Szira, F., Vágújfalvi, A., 2016. Relationship between SPAD value and grain yield can be affected by cultivar, environment and soil nitrogen content in wheat. Euphytica 211, 103–112. doi: 10.1007/s10681-016-1741-z

Monostori, I., Szira, F., Tondelli, A., Arendas, T., Gierczik, K., Cattivelli, L., Galiba, G., Vágújfalvi, A., 2017. Genome-wide association study and genetic diversity analysis on nitrogen use efficiency in a Central European winter wheat (*Triticum aestivum* L.) collection. PLoS One 12, e0189265. doi: 10.1371/journal .pone.0189265

Muchow, R.C., 1998. Nitrogen utilization efficiency in maize and grain sorghum. Field Crops Res. 56, 209–216. doi: 10.1016/S0378-4290(97)00132-9

Muurinen, S., Kleemola, J., Peltonen-Sainio, P, 2007. Accumulation and translocation of nitrogen in spring cereal cultivars differing in nitrogen use efficiency. Agron. J. 99, 441–449.

Namai, S., Toriyama, K., Fukuta, Y., 2009. Genetic variations in dry matter production and physiological nitrogen use efficiency in rice (*Oryza sativa* L.) varieties. Breed. Sci. 59, 269–276. doi: 10.1270/jsbbs.59.269

Nikolic, Ana, Andjelkovic, V., Dodig, D., Mladenović-Drinić, S., Kravić, N., Ignjatovic-Micic, D., 2013. Identification of QTL-s for drought tolerance in maize, II: Yield and yield components. Genetika 45, 141-150. doi:10.2298/GENSR1302341N.

Ortiz-Monasterio, J.I., Sayre, K.D., Rajaram, S., Mcmahon M., 1997. Genetic progress in wheat yield and nitrogen use efficiency under four nitrogen rates. Crop Sci. 37(3), 898–904. doi: 10.2135/cropsci1997.0011183X0037000 30033x

Panse, V.G., Sukhatme, P.V., 1967. Statistical methods for agricultural workers, ICAR Publication, New Delhi.

Paponov, I.A., Engels, C., 2005. Effect of nitrogen supply on carbon and nitrogen partitioning after flowering in maize. J. Plant. Nutr. Soil Sci. 168(4), 447-453.

Pathak, R.R., Lochab, S., Raghuram, N., 2011. Improving plant nitrogen-use efficiency. Comprehensive Biotechnology 4, 209-218. doi: 10.1016/B978-0-08-088504-9.00472-4

Pathak, R.R., Ahmad, A., Lochab, S., Raghuram, N., 2008. Molecular physiology of plant nitrogen use efficiency and biotechnological options for its enhancement. Curr. Sci. 94, 1394-1403.

Pramanik, K., Bera, A.K., 2013. Effect of seedling age and nitrogen fertilizer on growth, chlorophyll content, yield and economics of hybrid rice (*Oryza sativa* L.). Int. J. Agron. Plant Prod. 4, 3489–3499.

Presterl, T., Seitz, G., Landbeck, M., Thiemt, E.M., Schmidt, W., Geiger, H.H., 2003. Improving nitrogen-use efficiency in European maize. Crop Sci. 43, 1259–1265. doi: 10.2135/cropsci2003.1259

Pujarula, V., Pusuluri, M., Bollam, S., Das, R.R., Ratnala, R., Adapala, G., Thuraga, V., Rathore, A., Srivastava R.K., Gupta R., 2021. Genetic Variation for Nitrogen Use Efficiency Traits in Global Diversity Panel and Parents of Mapping Populations in Pearl Millet. Front. Plant Sci. 12, 625915. doi: 10.3389/fpls.2021.625915

Rangare, N.R., Krupakar, A., Kumar, A., Singh S.P., 2010. Character association and component analysis in wheat (*Triticum aestivum* L.). Electron. J. Plant Breed.1(3), 231-238.

Rao, I.S., Neeraja, C.N., Srikanth, B., Subrahmanyam, D., Swamy, K.N., Rajesh, K., Vijayalakshmi, P., Kiran, T.V., Sailaja, N., Revathi, P., Rao, P.R., Subba Rao, L.V., Surekha, K., Babu, V.R., Voleti, S.R., 2018. Identification of rice landraces with promising yield and the associated genomic regions under low nitrogen. Sci. Rep. 8, 9200. doi: 10.1038/s41598-018-27484-0

Raun, W.R., Johnson, G.V., 1999. Improving nitrogen use efficiency for cereal production. Agron. J. 91, 357–363.

Raun, W.R., Johnson, G.V., Westerman, R.L., 1999. Fertilizer nitrogen recovery in long-term continuous winter wheat. Soil Sci. Soc. Am. J., 63, 645–650. doi: 10.2136/sssaj1999.03615995006300030030x

Raun, W.R., Solie, J.B., Johnson, G.V., Stone, M.L., Mullen, R.W., Freeman, K.W., Thomason, W.E., Lukina, E.V., 2002. Improving nitrogen use efficiency in cereal grain production with optical sensing and variable rate application. J. Agron. 94, 815–820. doi: 10.2134/agronj2002.8150

Rengel, Z., Graham, R.D., 1995. Wheat genotypes differ in Zn efficiency when grown in chelate-buffered nutrient solution. Plant Soil 176, 307–316. doi: 10.1007/BF00011795

Robinson, H.F., Comstock, R.E., Harvey, P.H., 1949. Estimates of heritability and degree of dominance in corn. Agron. J. 41, 253- 259.

Rothstein, S.J., 2007. Returning to Our Roots: Making Plant Biology Research Relevant to Future Challenges in Agriculture. Plant cell 19(9), 2695-2699.

Russo, T.A., Tully, K., Palm, C., Neill, C., 2017. Leaching losses from Kenyan maize cropland receiving different rates of nitrogen fertilizer. Nutr. Cycling Agroecosyst. 108, 195–209. doi: 10.1007/ s10705-017-9852-z

Sadras, V.O., Lawson, C., 2013. Nitrogen and water-use efficiency of Australian wheat varieties released between 1958 and 2007. Eur. J. Agron.46, 34–41. doi: 10.1016/ j.eja.2012.11.008

Sarcevic, H., Jukic, K., Ikic, I., Lovric, A., 2014. Estimation of quantitative genetic parameters for grain yield and quality in winter wheat under high and low nitrogen fertilization. Euphytica 199, 57–67. doi: 10.1007/s10681-014-1154-9

Sial, M.A., Arain, M.A., Khanzada, S., Naqvi, M.H., Dahot, M.U., Nizamani, N.A., 2005. Yield and quality parameters of wheat genotypes as affected by sowing dates and high temperature stress. Pak. J. Bot. 37, 575–584.

Sivasubramanian, S., Menon, M., 1973. Heterosis and inbreeding depression in rice. Madras Agric. J. 60, 1139.

Snedecor, G.W., Cochran, W.G., 1989. Statistical Methods, Eighth Edition, Iowa State University Press.

Vijayalakshmi, P., Kiran, T.V., Rao, Y.V., Srikanth, B., Rao, I.S., Sailaja, B., Surekha, K., Rao, P.R., Subrahmanyam, D., Neeraja, C.N., Voleti, S.R., 2013. Physiological approaches for increasing nitrogen use efficiency in rice. Indian J. Plant Physiol. 18, 208–222. doi: 10.1007/s40502-013-0042-y

Vijayalakshmi, P., Kiran, T.V., Kumari, B.R., Srikanth, B., Rao, I.S., Swamy, K.N., Surekha, K.,Sailaja, N., Subbarao, L.V., Rao, P.R., Subrahmanyam, D., Neeraja, C.N., Voleti, S.R., 2015. Biochemical and physiological characterization for nitrogen use efficiency in aromatic rice genotypes. Field Crops Res. 179, 132–143. doi:0.1016/j.fcr.2015.04.012

Vitousek, P.M., Mooney, H.A., Lubchenco, J., Melillo, J.M., 1997. Human domination of Earth's ecosystems. Sci. 277, 494–499. doi: 10.1126/science. 277.5325.494

Worku, M., Bänziger, M., Erley, G.S.A.M., Friesen, D., Diallo, A.O., Horst, W. J., 2007. Nitrogen uptake and utilization in contrasting nitrogen efficient tropical maize hybrids. Crop Sci. 47, 519–528. doi: 10.2135/cropsci2005.05.0070

Würschum, T., 2012.Mapping QTL for agronomic traits in breeding populations. Theor. Appl. Genet. 125, 201–210. doi: 10.1007/s00122-012-1887-6

Yang, H., Yang, J., Lu, Y., He, J., 2014. SPAD values and nitrogen nutrition index for the evaluation of rice nitrogen status. Plant Pro. Sci. 17, 81–92. doi: 10.1626/pps.17.81

Yousaf, A., Atta, B. M., Akhter, J., Monneveux, P., Lateef, Z., 2008, Genetic variability, association and diversity studies in wheat (*T. aestivum*) germplasm. Pak. J. Bot. 40, 2087-2097.

Zadoks, J.C., Chang, T.T., Konzak, C.F., 1974. Decimal code for growth stages of cereals. Weed Res. 14, 415–421.

Zafar, J., Muhammad, F.C., 2007. Effects of soil and foliar application of different concentrations of NPK and foliar application of $(NH_4)_2SO_4$ on growth and yield attributes in wheat (*Triticum aestivum* L). Pak. J. Agric. Sci.13(2), 119-128.

Zhang, X., Davidson, E.A., Mauzerall, D.L., Searchinger, T.D., Dumas, P., Shen, Y., 2015. Managing nitrogen for sustainable development. Nature 528, 51–59.

Fig.1. The relationship between grain yield and NUE under two nitrogen levels

Click here to access/download;Figure;Fig. 1 NUE Plos one.tiff

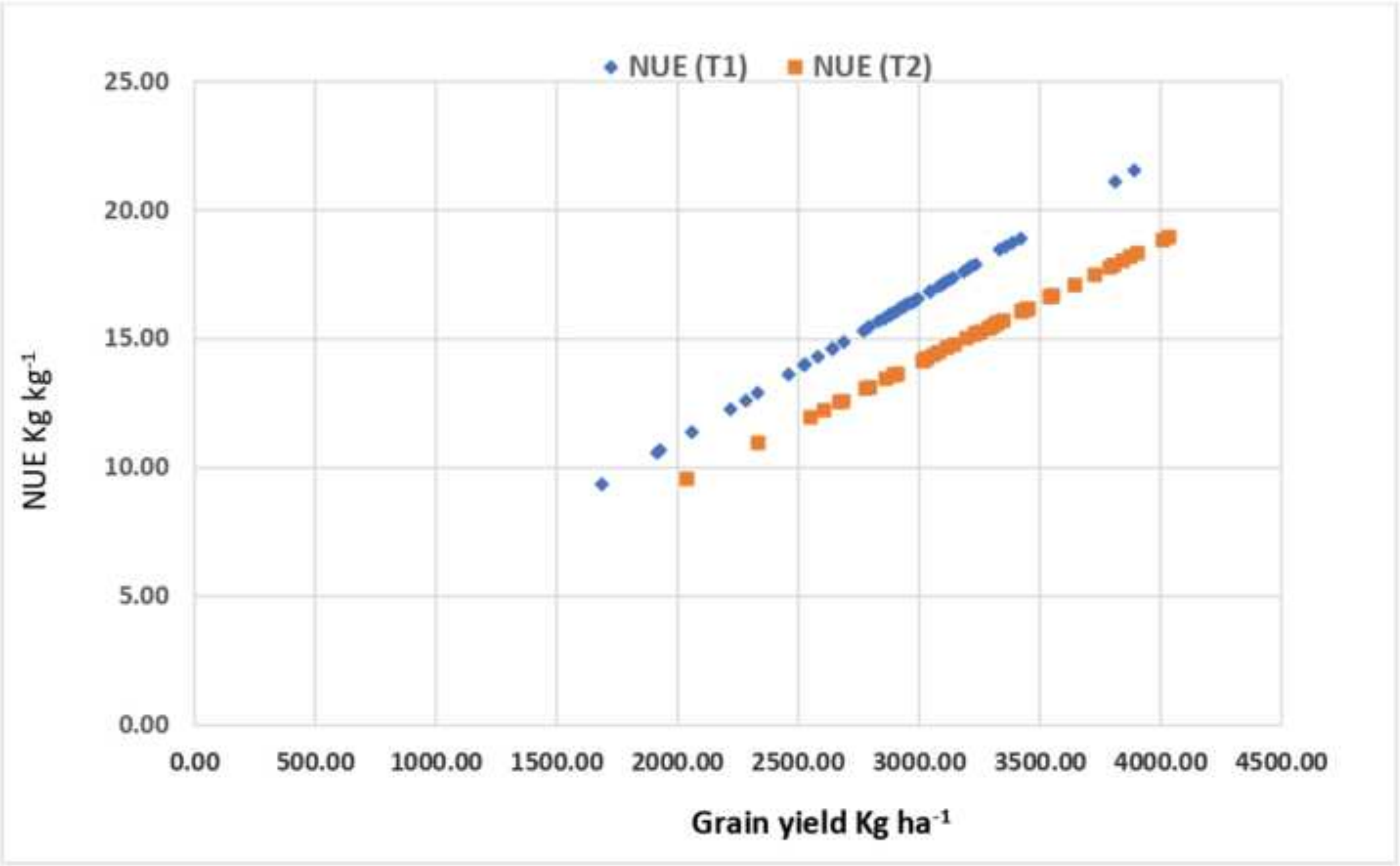

Fig 2A: Top ten high grain yielding genotypes T1 (N50) and T1 (N100) Click here to access/download;Figure;Fig 2a NUE PLos one.tiff

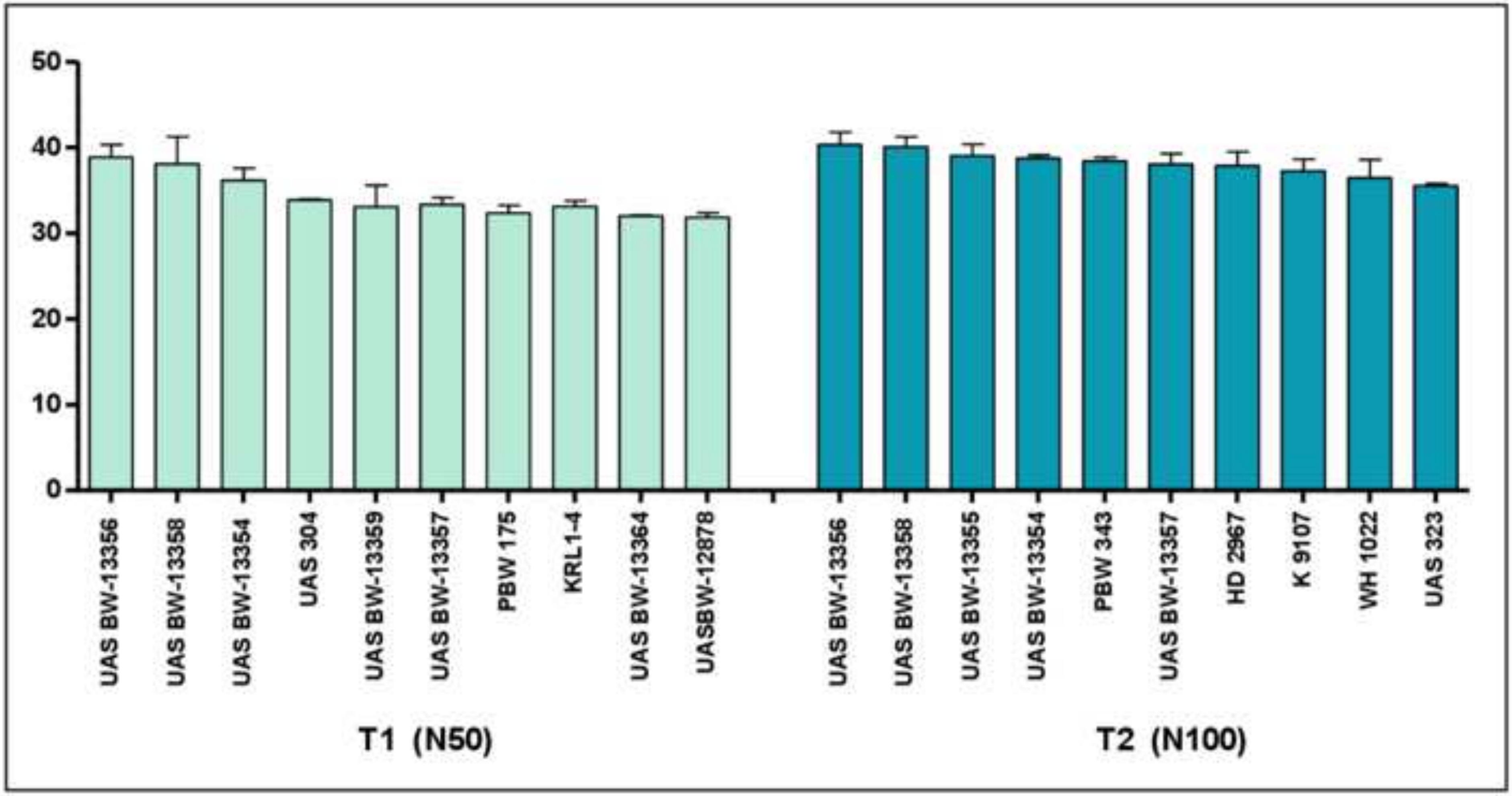

Fig 2B: Top ten poor grain yielding genotypes T1 (N50) and T1 (N100)

Click here to access/download;Figure;Fig 2b NUE PLoS One.tiff

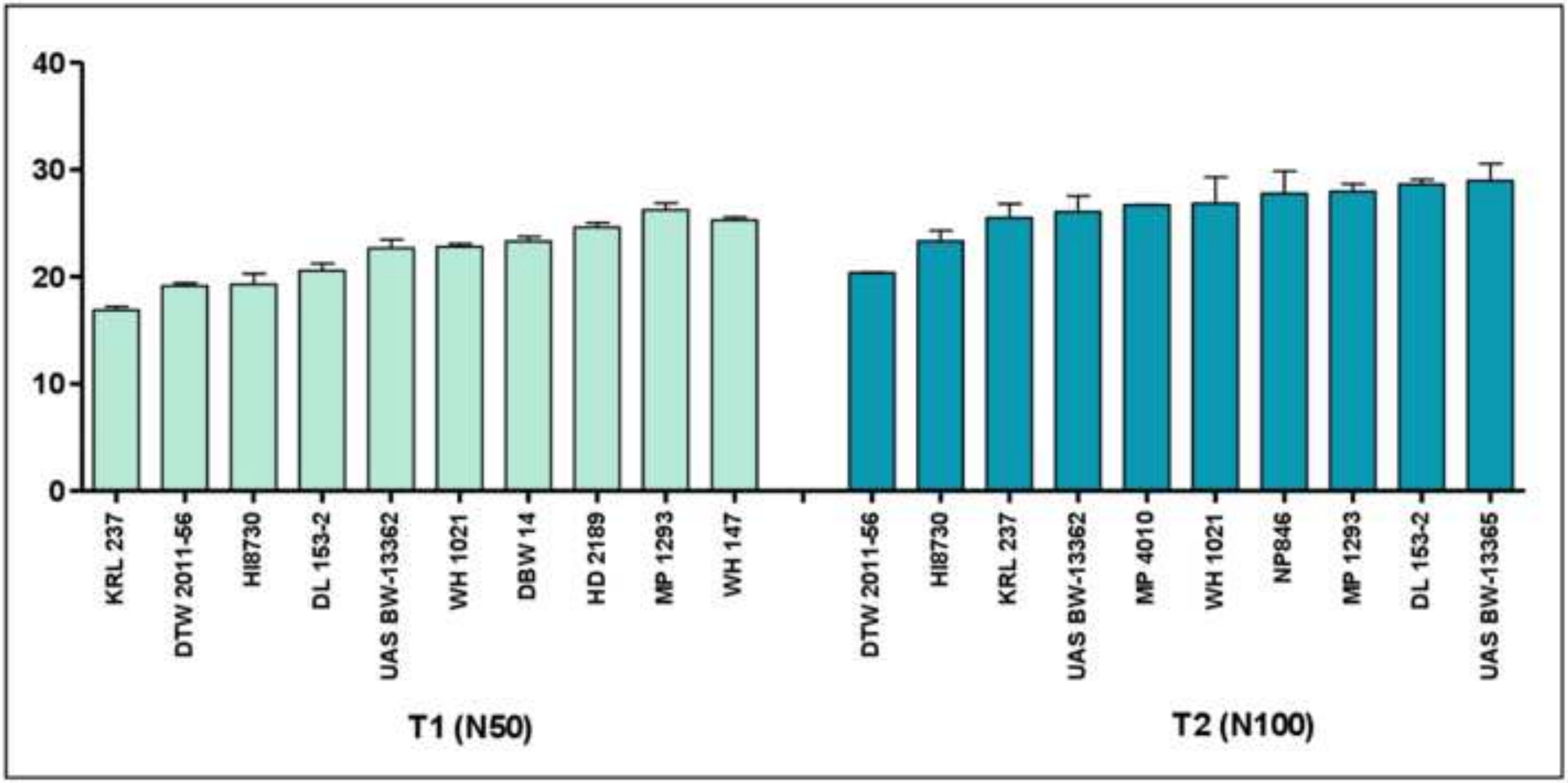

Fig.3: Relationship between genotypic performance for NUtE under low (T1- N50) and recommended (T2-N100) N conditions. (The horizontal line represents the mean NUtE

Click here to access/download;Figure;Fig 3 NUE PLoS one.tiff

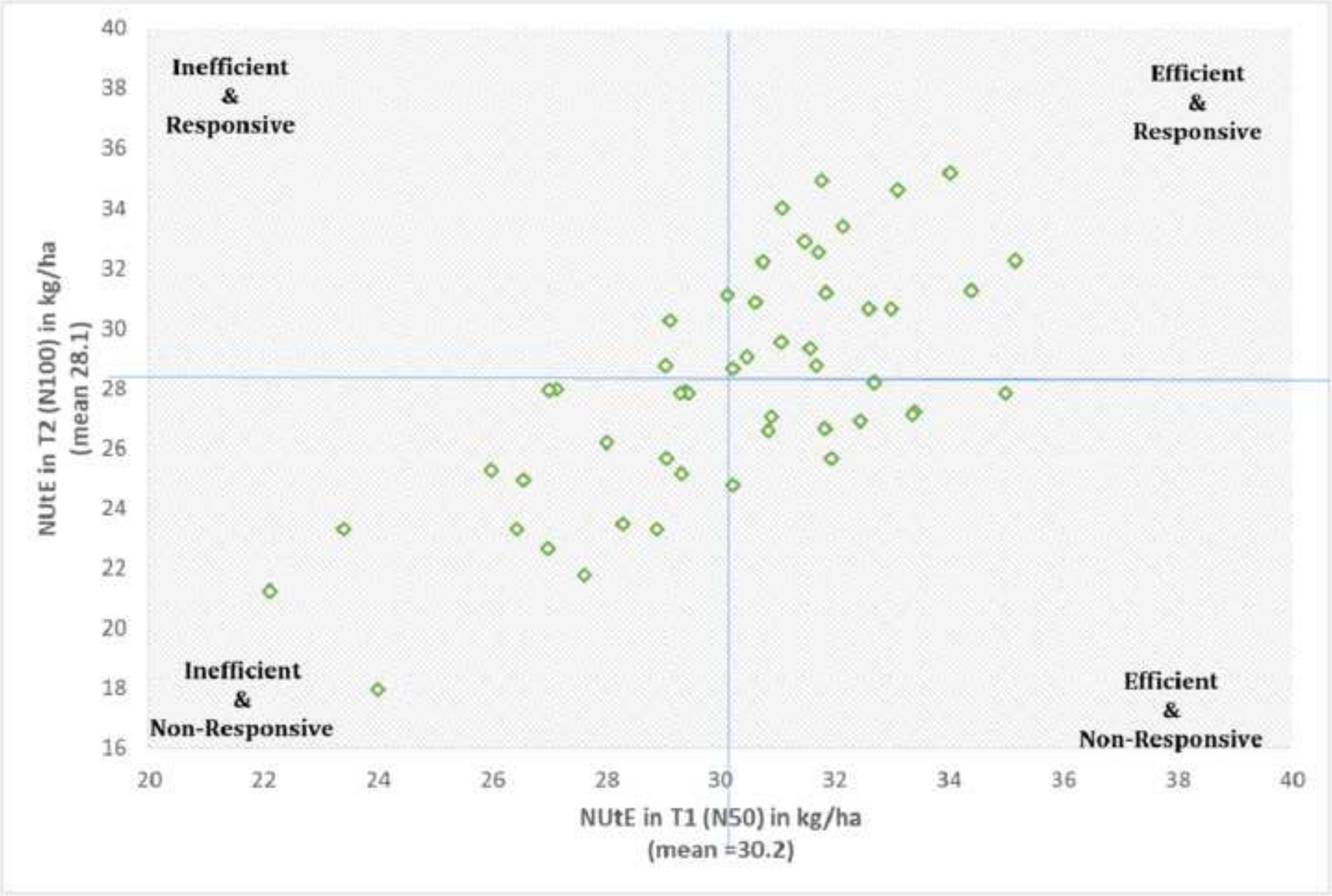

Fig.4: Frequency distribution for various NUE related traits in wheat under T1 and T2 conditions

Click here to access/download;Figure;Fig. 4 NUE Plos one.tiff

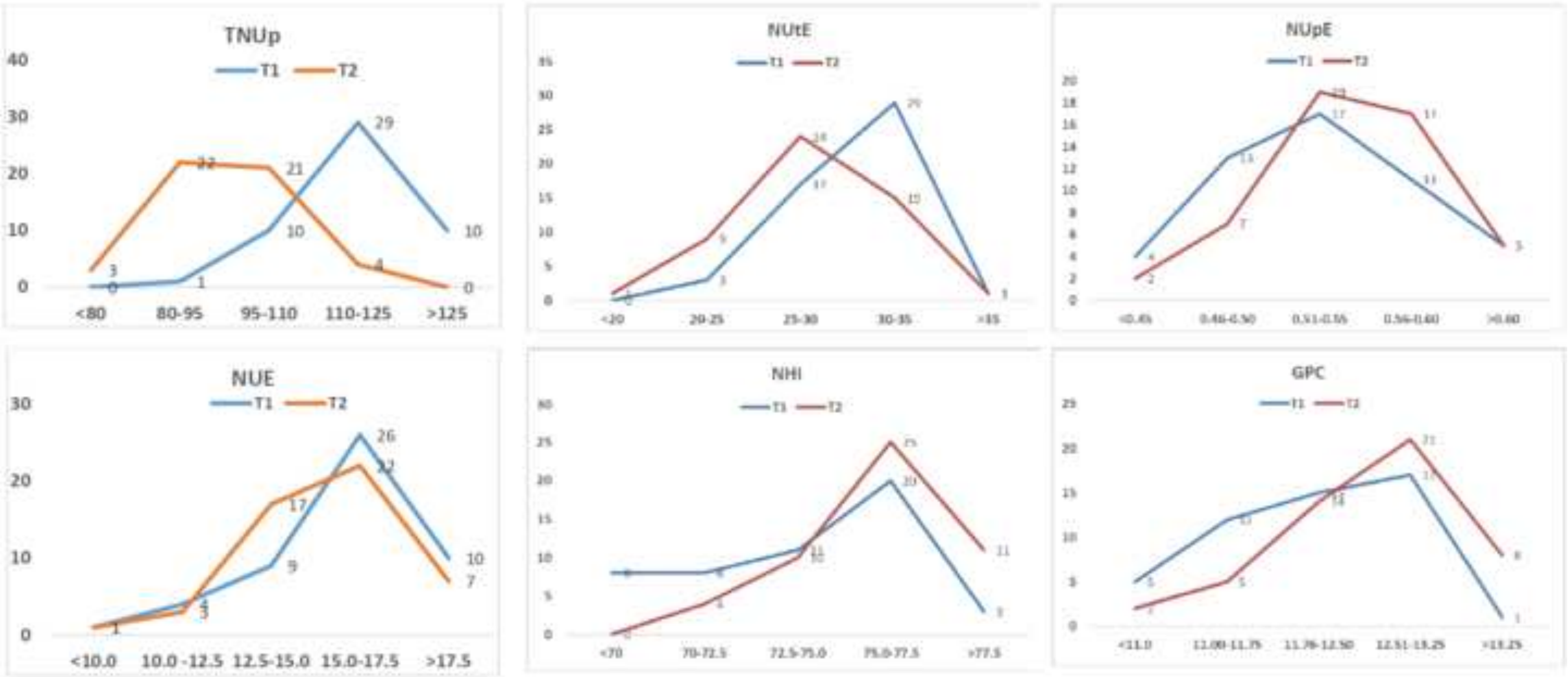

Supplement: Attachment — Submitted filename: PONE-D-23-04727 (1).pdf [file pone.0294755.s007.pdf]
