# Supplementary material for: Nitrogen use efficiency in bread wheat: Genetic variation and prospects for improvement
Source: PLoS One. 2024 Apr 10;19(4):e0294755. doi: 10.1371/journal.pone.0294755 (PMC11006162; doi:10.1371/journal.pone.0294755)
Supplement: Attachment — Submitted filename: Responce to Reviewers.docx [file pone.0294755.s010.docx]

REVIEWERS COMMENTS

NEEDS MAJOR REVISION PRIOR ACCPETANCE

1. There are many grammatical errors of prepositions, verbs etc. Please check the pdf file attached for detailed corrections

**Corrections are made in the manuscript and also highlighted in the track-changes version of the Manuscript**

2. Have you added any supplementary file along with the main manuscript? As under methodology section, like in case of Table 3, you have mentioned few traits, the tables containing values for the rest of traits are missing.

**All traits added in Supplementary table and Corrections are made in the manuscript**

3. Statistical part is fine but please also refer to this article for statistical part analysis Ivić M, Grljušić S, Plavšin I, Dvojković K, Lovrić A, Rajković B, Maričević M, Černe M, Popović B, Lončarić Z, Bentley AR, Swarbreck SM, Šarčević H and Novoselović D (2021) Variation for Nitrogen Use Efficiency Traits in Wheat Under Contrasting Nitrogen Treatments in South-Eastern Europe. Front. Plant Sci. 12:682333. doi: 10.3389/fpls.2021.682333.

**Few extra analyses were done as per the suggested article and other PLos one articles and incorporated in the manuscript.**

4. Add latest references of 2022-2023 as well related to nitrogen use efficiency in wheat.

**Added recent references of 2022-23 in the Manuscript.**

5. Check for the spelling of this reference in the text. Choukan, R., Taherkhani, T., Ghanadha, M.R., Khodarahmi, M., 2006. Evaluation of drought tolerance in grain maize inbred lines using drought tolerance indices. Iranian Journal of Crop Sci. 8(1), 79-89

**Correction is made in the manuscript and also highlighted in the track-changes version of the Manuscript.**
